# Inhibition of SARS-CoV-2 main protease by phenolic compounds from *Manilkara hexandra* (Roxb.) Dubard assisted by metabolite profiling and *in silico* virtual screening†

**DOI:** 10.1039/d0ra05679k

**Published:** 2020-08-28

**Authors:** Fatma M. Abd El-Mordy, Mohamed M. El-Hamouly, Magda T. Ibrahim, Gehad Abd El-Rheem, Omar M. Aly, Adel M. Abd El-kader, Khayrya A. Youssif, Usama Ramadan Abdelmohsen

**Affiliations:** Department of Pharmacognosy, Faculty of Pharmacy (Girls), Al-Azhar University 11754 Cairo Egypt; Department of Pharmacognosy, Faculty of Pharmacy (Boys), Al-Azhar University 11371 Cairo Egypt; Department of Pharmacognosy, Faculty of Pharmacy, Sinai University 41636 Kantara Branch Egypt; Department of Pharmacology, National Research Centre 12622 Giza Egypt; Department of Medicinal Chemistry, Faculty of Pharmacy, Minia University 61519 Minia Egypt; Department of Pharmacognosy, Faculty of Pharmacy, Deraya University 61111 Minia Egypt Usama.ramadan@mu.edu.eg; Department of Pharmacognosy, Faculty of Pharmacy, Al-Azhar University Assiut 71524 Egypt; Department of Pharmacognosy, Faculty of Pharmacy, Modern University for Technology and Information Cairo Egypt; Department of Pharmacognosy, Faculty of Pharmacy, Minia University 61519 Minia Egypt

## Abstract

SARS-CoV-2 is a novel coronavirus that was first identified during the outbreak in Wuhan, China in 2019. It is an acute respiratory illness that can transfer among human beings. Natural products can provide a rich resource for novel antiviral drugs. They can interfere with viral proteins such as viral proteases, polymerases, and entry proteins. Several naturally occurring flavonoids were reported to have antiviral activity against different types of RNA and DNA viruses. A methanolic extract of *Manilkara hexandra* (Roxb.) Dubard leaves is rich in phenolic compounds, mainly flavonoids. Metabolic profiling of the secondary metabolites of *Manilkara hexandra* (Roxb.) Dubard leaves methanolic extract (MLME), and bark ethyl acetate (MBEE) extract using LC-HRESIMS resulted in the isolation of 18 compounds belonging to a variety of constituents, among which phenolic compounds, flavones, flavonol glycosides and triterpenes were predominant. Besides, four compounds (I–IV) were isolated and identified as myricetin I, myricitrin II, mearnsitrin III, and mearnsetin-3-*O*-β-d-rutinoside IV (compound IV is isolated for the first time from genus *Manilkara*) and dereplicated in a metabolomic study as compounds 3, 5, 6, and 12, respectively. The molecular docking study showed that rutin, myricitrin, mearnsitrin, and quercetin 3-*O*-β-d-glucoside have strong interaction with SARS-CoV-2 protease with high binding energy of −8.2072, −7.1973, −7.5855, and −7.6750, respectively. Interestingly, the results proved that rutin which is a citrus flavonoid glycoside exhibits the strongest inhibition effect to the SARS-CoV-2 protease enzyme. Consequently, it can contribute to developing an effective antiviral drug lead against the SARS-CoV-2 pandemic.

## Abstract 2

SARS-CoV-2 is a novel coronavirus that was first identified during the outbreak in Wuhan, China in 2019.

## Graphical abstract

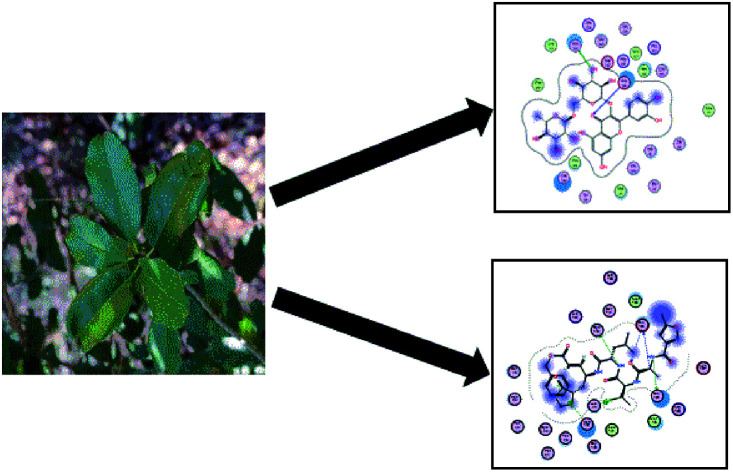

## 1. Introduction

The SARS-CoV-2 virus is the main cause of the 2019–2020 viral pneumonia outbreak that appeared first in Wuhan.^1–4^ Finding a known drug that inhibits the SARS-CoV-2 virus main protease (M^pro^) will have a pivotal role in controlling viral replication and transcription.^5,6^ Zhenming Jin, *et al.*, determined the crystal structure of SARS-CoV-2 virus M^pro^ in complex with N3 mechanism-based inhibitor.^7^ The functional polypeptides are released from the polyproteins by extensive proteolytic processing, predominantly by a 33.8 kDa main protease (M^pro^), also referred to as the 3C-like protease. M^pro^ digests the polyprotein at no less than 11 conserved sites, starting with the autolytic cleavage of this enzyme itself from pp1a and pp1ab.^8^ The functional importance of M^pro^ in the viral life cycle, together with the absence of closely related homologs in humans, identify M^pro^ as an attractive target for antiviral drug design.^9^ Several naturally occurring flavonoids possess variable antiviral activity against certain RNA viruses such as respiratory syncytial virus (RSV), parainfluenza virus type 3 (Pf-3), polio-virus type 1 (polio) and DNA viruses such as Herpes Simplex Virus type 1 (HSV-1).^10^ Antiviral activity of the myricetin derivatives and methoxy flavones was evaluated against Hepatitis B Virus (HBV), Herpes Simplex Virus type 1 (HSV-1), and Poliovirus type 1 (PV-1); it showed antiviral activity without cytotoxic effects. The methoxy flavones were the most active compounds, showing an antiviral effect against all the evaluated viruses.^11^ Therefore, this study aimed to estimate the total phenolic and flavonoid content, metabolic profiling with LC-HRESIMS-assisted chemical investigation of the secondary metabolites of *Manilkara hexandra* (Roxb.) Dubard (family: Sapotaceae), isolation, and structure elucidation of four flavonol compounds. Additionally, a molecular docking study was performed to study the inhibitory effect of these flavonoids against SARS-CoV-2 main protease enzymes.

## 2. Results and discussion

### 2.1 Metabolic profiling

Metabolomics is a valuable and comprehensive analysis tool that is used to study the metabolite profiles of unicellular and multicellular biological systems.^14^ Plants represent a major challenge in metabolomics due to the high chemical diversity of their metabolites.^14^ Consequently, no single analytical method can determine all plant metabolites simultaneously, but LC-HRESIMS is a powerful analytical tool for metabolic profiling that can detect/dereplicate a wide range of chemical compounds at the same time without tedious isolation procedures.^15,16^

**Fig. 1:**
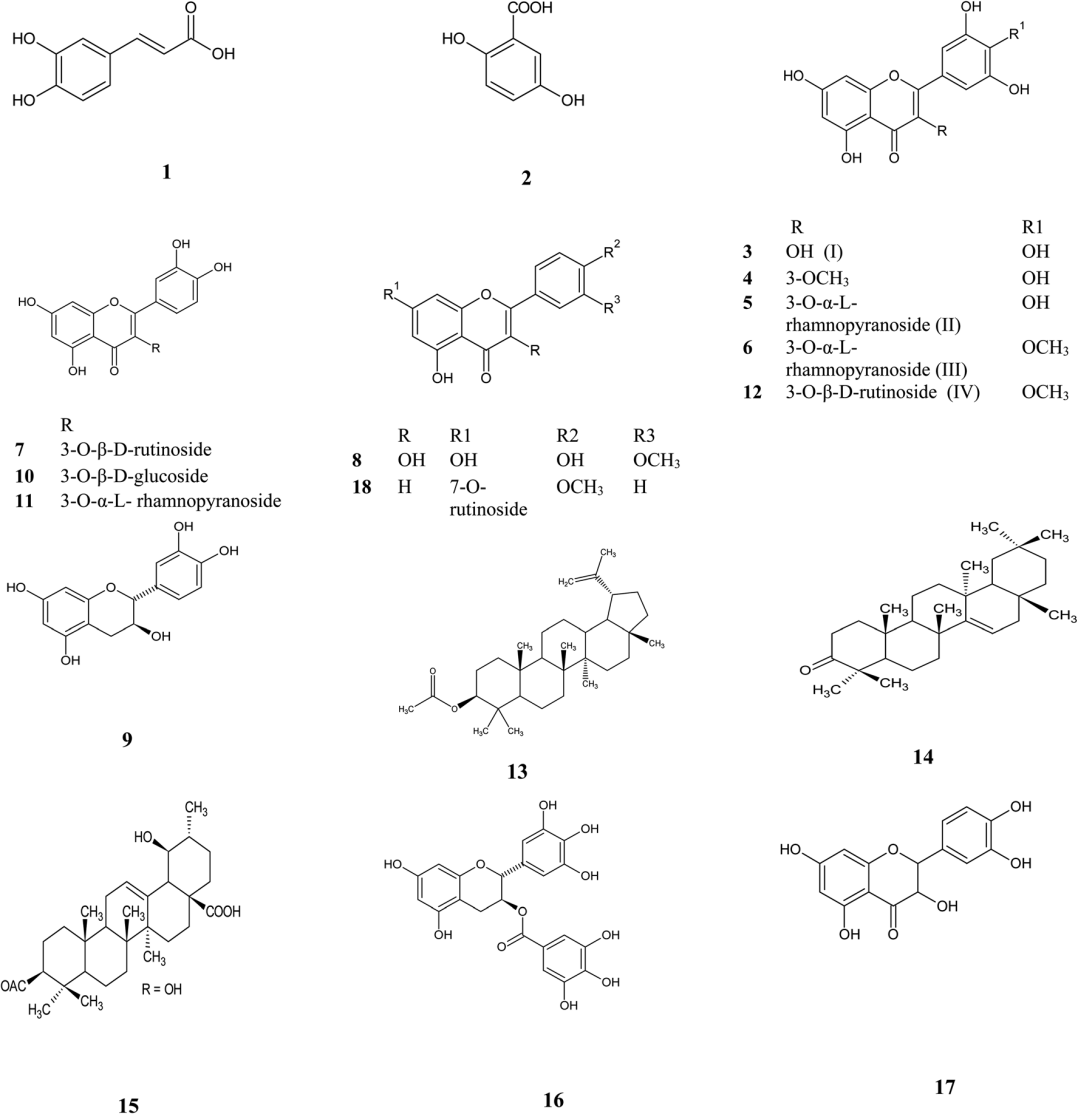
Isolated and annotated metabolites from the LC-HRESIMS analysis of *Manilkara hexandra* (Roxb.) Dubard leaves and bark extracts.

Metabolic profiling of the secondary metabolites of *Manilkara hexandra* (Roxb.) Dubard leaves methanolic extract (MLME), and bark ethyl acetate (MBEE) extract using LC-HRESIMS (ESI, Fig. S18†) resulted in the annotation of eighteen compounds belong to a variety of constituents, among which phenolic, flavones, flavonol glycosides and triterpenes were predominant,^12,13^ with qualitative and quantitative variation in each extract (Table 1 and Fig. 1).

**Table 1:** The LC-HR-ESIMS annotation results of *Manilkara hexandra* (Roxb.) Dubard leaves and bark extracts^a^

No.	Metabolites name	Original source	Chemical class	Mode	MF	RT (min)	*m*/*z*	References
1	Caffeic acid	*Manilkara zapota*	Phenolic acid	+	C_9_H_8_O_4_	3.27	181.0489	17
2	2,5-Dihydroxy benzoic acid	*Alchornea cordifolia*	Unsaturated carboxylic acid	+	C_7_H_6_O_4_	2.11	155.0332	18
3	Myricetin I	*Mimusops laurifolia*	Flavonol	+	C_15_H_10_O_8_	2.65	319.0441	21
4	Myricetin-3-*O*-methyl ether	*Pteroxygonum giraldii*	Flavonol	+	C_16_H_12_O_8_	2.97	333.0608	19
5	Myricitrin II	*Manilkara Subsericea*	Flavonol glycoside	+	C_21_H_20_O_12_	4.31	465.1026	19
6	Mearnsitrin III	*Mimusops laurifolia*	Flavonol glycoside	+	C_22_H_22_O_12_	4.51	479.1179	21
7	Rutin	*Mimusops laurifolia*	Flavonol glycoside	+	C_27_H_30_O_16_	1.88	611.1604	21
8	3′-Methoxy-4′,5,7-trihydroxy flavonol	*Cuscuta reflexa*	Flavonol	+	C_16_H_12_O_7_	3.81	317.0653	22
9	(+) Catechin	*Manilkara zapota*	Flavan-3-ol	+	C_15_H_14_O_6_	2.49	291.0787	25
10	Quercetin 3-*O*-β-d-glucoside	*Mimusops laurifolia*	Flavonol glycoside	+	C_21_H_20_O_12_	3.38	465.1026	21
11	Quercetin 3-*O*-α-l-rhamnopyranoside	*Manilkara Subsericea*	Flavonol glycoside	+	C_21_H_20_O_11_	2.69	449.1073	41
12	Mearnsetin-3-*O*-β-d-rutinoside IV	*Manilkara hexandra*	Flavonol glycoside	+	C_28_H_32_O_17_	5.55	641.1713	21
13	Lupeol-3-acetate	*Mimusops elengi*	Triterpene	+	C_32_H_52_O_2_	4.32	469.4034	28
14	Taraxerone	*Mimusops elengi*	Triterpene	+	C_30_H_48_O	5.62	425.3785	42
15	3-Acetyl ursolic acid	*Mimusops obtusifolia*	Triterpene	+	C_32_H_50_O_4_	4.04	499.3736	27
16	Gallocatechin-3-*O*-gallate	*Camellia sinensis*	Catechin	+	C_22_H_18_O_11_	6.35	459.0846	26
17	Taxifolin	*Mimusops elengi*	Flavanonol	+	C_15_H_12_O_7_	2.86	305.0657	24
18	Acacetin-7-*O*-rutinoside	*Origanum syriacum*	Flavonoid-7-*O*-glycoside	+	C_28_H_32_O_14_	3.39	593.1873	23

a MF: molecular formula, RT: retention time, min: minute.

From the metabolomics data, the mass ion peak at *m*/*z* 181.0489 for the predicted molecular formula C_9_H_8_O_4_ was dereplicated as caffeic acid, which was previously detected in *Manilkara zapota* and first reported in *Manilkara hexandra* (Roxb.) Dubard,^17^ whereas that at *m*/*z* 155.0332, corresponding to the suggested molecular formula C_7_H_6_O_4_ was dereplicated as 2,5 dihydroxy benzoic acid, which was formerly reported from the African tree *Alchornea cordifolia* and first reported from *Manilkara* genus.^18^ Likewise, a flavone aglycon with the molecular formula C_15_H_10_O_8_ was characterized as 3,5,7,3′,4′,5′-hexahydroxy-flavone (myricetin) I from the mass ion peak at *m*/*z* 319.0441, which was previously obtained from *Mimusops laurifolia*.^19^ Moreover, the mass ion peak at *m*/*z* 333.0608, in agreement with the predicted molecular formula C_16_H_12_O_8_ was dereplicated as myricetin-3-*O*-methyl ether. This has been isolated earlier from *Pteroxygonum giraldii*.^20^ The mass ion peaks at *m*/*z* 465.1026 and 479.1179, for the predicted molecular formulas C_21_H_20_O_12_, C_22_H_22_O_12_, and were dereplicated as the flavonol glycoside myricetin 3-*O*-α-l-1C4 rhamnopyranoside (myricitrin) II and myricetin-4′-*O*-methyl ether-3-*O*-α-l-rhamnopyranoside (mearnsitrin) III, respectively, which were previously detected in *Manilkara Subsericea* and *Mimusops laurifolia* respectively, and were first reported in *Manilkara hexandra* (Roxb.) Dubard.^19,21^ Whereas, the mass ion peaks at *m*/*z* 641.1713, corresponding to the suggested molecular formula C_28_H_32_O_17_, was identified as mearnsetin-3-*O*-β-d-rutinoside IV, which was the first report for this metabolite in *Manilkara* genus and *Manilkara hexandra* (Roxb.) Dubard. Likewise, another flavonol glycosides with the molecular formulas C_27_H_30_O_16_, C_21_H_20_O_12_ and C_21_H_20_O_11_ were characterized as quercetin-3-*O*-β-d-rutinoside (rutin), quercetin 3-*O*-β-d-glucoside and quercetin 3-*O*-α-l-rhamnopyranoside (quercitrin), from the mass ion peak at *m*/*z* 611.1604, 465.1026 and 449.1073, were previously obtained from *Mimusops laurifolia* and *Manilkara Subsericea*.^19,21^ Additionally, a compound at *m*/*z* 317.0653, corresponding to the suggested molecular formula C_16_H_12_O_7_ was dereplicated as 3′-methoxy-4′,5,7 trihydroxy flavonol, which was formerly reported from the species *Cuscuta reflexa* and was first reported in the *Manilkara* genus.^22^ Likewise, a flavonoid-7-*O*-glycosides compound with the molecular formula C_28_H_32_O_14_ was characterized as acacetin-7-*O*-rutinoside from the mass ion peak at *m*/*z* 593.1873 and was previously obtained from *Origanum syriacum*.^23^ Furthermore, a compound at *m*/*z* 305.0657, corresponding to the suggested molecular formula C_15_H_12_O_7_ was dereplicated as taxifolin (5,7,3′,4′-flavan-on-ol), which was formerly reported from the *Mimusops elengi*.^24^ Another mass ion peaks at *m*/*z* 291.0787 and 459.0846 for the predicted molecular formula C_15_H_14_O_6_ and C_22_H_18_O_11_, respectively was dereplicated as (+) catechin and, gallocatechin-3-*O*-gallate respectively, the two compounds were previously detected in *Manilkara zapota* and *Camellia sinensis*,^25,26^ and first reported in *Manilkara hexandra* (Roxb.) Dubard. In addition to the above-mentioned molecules, triterpenoid compounds at the mass ion peaks at *m*/*z* 469.4034, 425.3785 and 499.3736 for the suggested molecular formulas C_32_H_52_O_2_, C_30_H_48_O and C_32_H_50_O_4_ were annotated as lupeol-3-acetate, taraxerone, and 3-acetyl ursolic acid, respectively, which were previously reported in *Mimusops elengi* and *Mimusops obtusifolia*.^27,28^

### 2.2 Structure elucidation of the isolated compounds

Based on the physicochemical, chromatographic properties, and spectral analysis using (UV, MS, ^1^H-NMR and ^13^C-NMR), as well as comparison with the literature and some authentic samples, four compounds were isolated and identified as 3,5,7,3′,4′,5′-hexahydroxy-flavone (myricetin) (I),^29^ myricetin 3-*O*-α-l-1C4 rhamnopyranoside (myricitrin) (II),^30^ myricetin-4′-*O*-methyl ether-3-*O*-α-l-rhamnopyranoside (mearnsitrin) (III),^19^ and mearnsetin-3-*O*-β-d-rutinoside (IV).^19^ Compounds I–III were previously isolated from *Manilkara* species, but they were isolated for the first time from the species *Manilkara hexandra* (Roxb.) Dubard.^17,19,21^ Compound IV was isolated for the first time from *Manilkara* species as well as *Manilkara hexandra* (Roxb.) Dubard.

### 2.3 Anti-SARS-CoV-2 molecular docking study

**Fig. 2:**
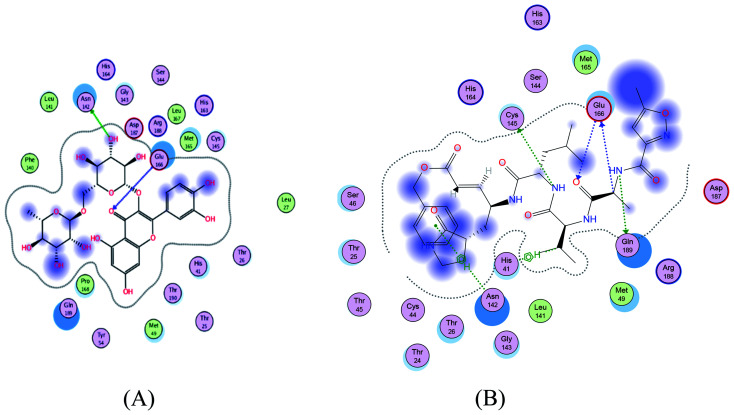
2D representation of docking of compounds 7 (A), and N3 (B) into the N3 binding site of the SARS-CoV-2 main protease.

**Fig. 3:**
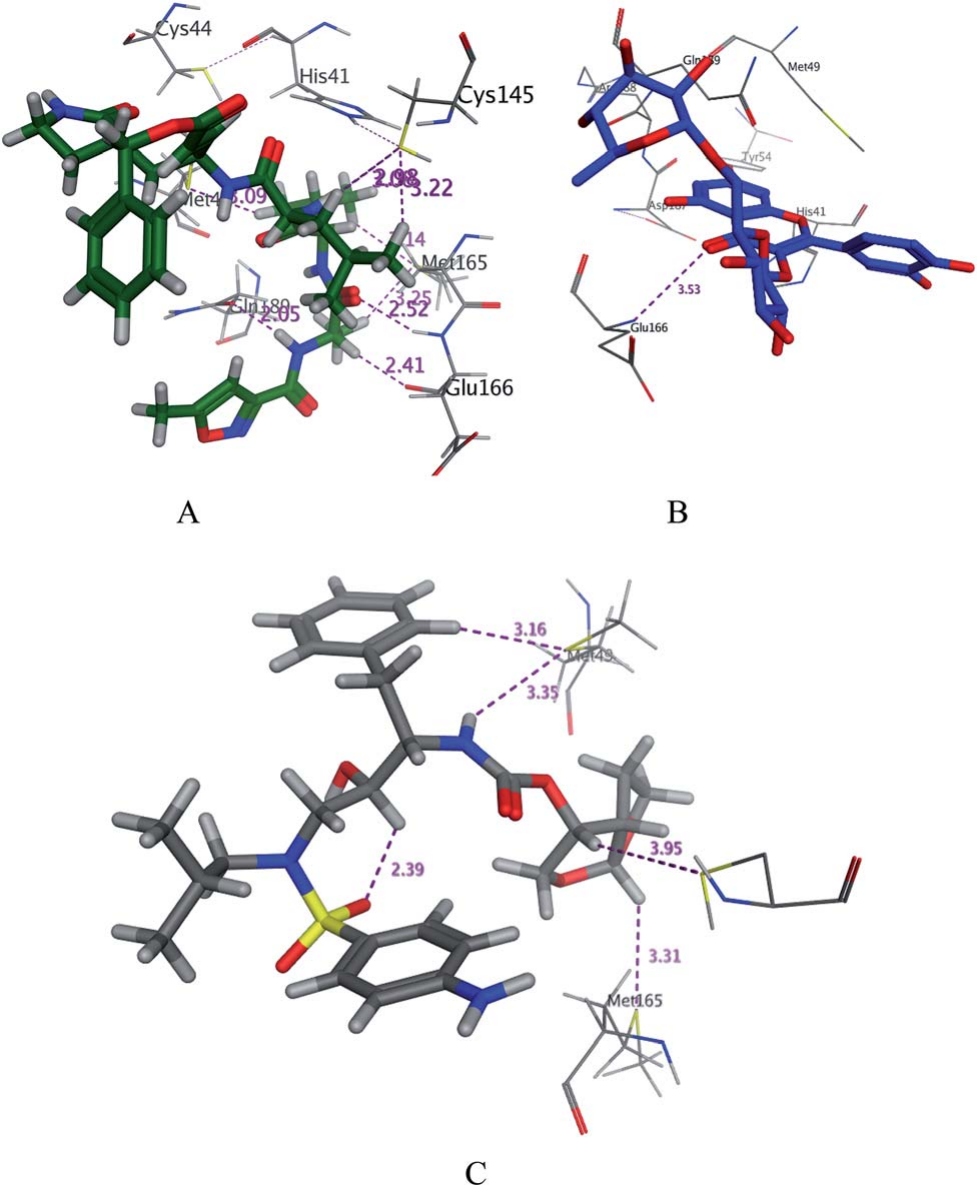
3D docking poses of compounds 7 (A), and N3 (B) and darunavir (C) into the N3 binding site of the SARS-CoV-2 main protease.

**Fig. 4:**
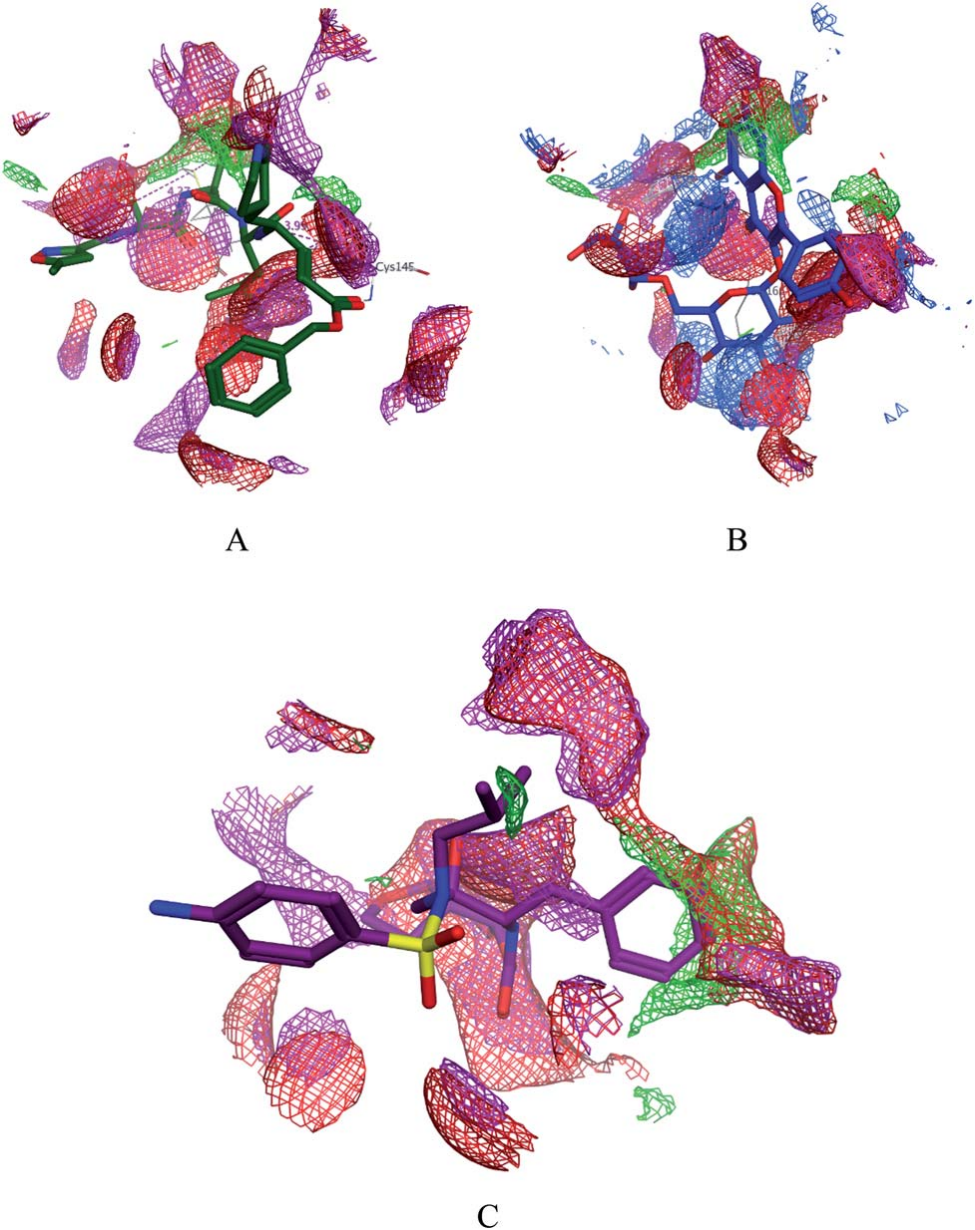
Surface and maps of the interaction potential of compounds 7 (A) and N3 (B) and darunavir (C) with the N3 binding site of the SARS-CoV-2 main protease.

SARS-CoV-2 virus M^pro^ has a Cys–His catalytic dyad, and the substrate-binding site is located in a cleft between domain I and II. A Michael acceptor inhibitor known as N3 was developed using computer-aided drug design.^31,32^ N3 is fitted inside the substrate-binding pocket of SARS-CoV-2 virus M^pro^ showing asymmetric units containing only one polypeptide. All compounds showed nearly bindings like N3. Results of interaction energies with M^pro^ are shown in Table 2. Molecular docking simulation of the compounds, darunavir and N3 into M^pro^ active site was performed. They got stabilized at the N3-binding site of M^pro^ by variable several electrostatic bonds (Fig. 2–4). The order of strength of binding was as follows:N3> 7 > 10 > 6 > 5 > darunavir > 11 > 4 > 3 > 9 > 8 > 1 > 2

**Table 2:** The receptor interaction of and the binding energy scores of the identified compounds, darunavir, and N3 into the N3 binding site in the SARS-CoV-2 main protease

Compound	dG, kcal mol^−1^	E_Conf., kcal mol^−1^	E_Place, kcal mol^−1^	E_Refine	Receptor
				dG, kcal mol^−1^	Amino acid/type of bonding/distance (Å)/binding energy (kcal mol^−1^)
1	−5.1104	−20.7880	−7.4002	−8.2307	Met49/H-donor/3.51/−0.2
2	−3.6132	31.6316	−15.5250	−17.3981	—
3 (I)	−5.9328	31.6316	−15.5250	−17.3981	Met165/H-donor/3.26/−0.4
4	−6.2258	48.0913	−15.3310	−19.7152	Gln189/Pi-H/4.25/−0.7
5 (II)	−7.1973	122.4130	−18.0611	−21.2239	Met165/H-donor/2.71 and 3.28/−0.3 and −0.2 and Cys145/H-donor/3.68/−0.2
6 (III)	−7.5855	145.0424	−19.1410	−26.5002	Phe140/H-donor/2.93/−1.8 and Gln189/H-donor/3.29/−0.7, Met49/H-donor/3.58/−1.0
7	−8.2072	211.5644	−24.6113	−30.4412	Asn142/H-donor/3.05/−2.2 and Glu166/H-acceptor 3.53/−0.8
8	−5.7864	62.4023	−11.8787	−17.1581	Thr190/H-donor/2.25/−0.4
9	−5.8134	23.4276	−12.6033	−18.7543	His164/H-donor/2.56/−0.3, Met165/H-donor/3.75/−1.6 and Gln189/H-donor/2.47/−0.2
10	−7.6750	126.5861	−15.3358	−25.6531	Met165/H-donor/3.65/−0.4 and His163/H-acceptor 3.09/−2.3
11	−6.9873	128.	−21.1809	−20.2922	Met165/H-donor/1.75/−1.5, Met49/H-donor/1.95/−0.99, Cys145/H-donor/1.81/−0.51, Gln189/H-donor/2.01/−0.63 and His 163/H-donor/2.4/−0.41
Darunavir	−7.0415	−26.4826	−18.8722	−12.8003	Cys145/H-donor/3.95/−0.4, Met49/H-donor/3.35/−0.7, Gln189/H-donor/2.2/−0.42 and His41/H-Pi/3.33/−0.1
N3	−8.4767	−4.5013	−18.6098	−24.9301	Gln189/H-donor 2.96/−2.3, Glu166/H-donor/3.34/−0.6, Cys145/H-donor/3.99/−2.0, Glu166/H-acceptor 3.48/−0.6, His41/H-Pi/3.71/−0.7 and Asn/Pi-H/4.16/−1.2

The glycosidic compounds were promising as protease inhibitors than the other aglycone phenolic compounds. Rutin was the most promising compound with high binding energy in comparison with the other compounds.

It worth mentioning that several molecular docking studies were performed to systematically investigate the effect of flavonoids on SARS-CoV-2 target proteins.^33,34^ Owis *et al.*^35^ reported that flavonoid compounds narcissin, kaempferol-3-*O*-α-l-rhamnopyranosyl-(1→6)-β-d-glucopyranoside, isorhamnetin-3-*O*-α-l-rhamnopyranosyl-(1→6)-*O*-[α-l-rhamnopyranosyl-(1→2)]-*O*-β-d-glucopyranoside, and isorhamnetin-3-*O*-α-l-rhamnopyranosyl-(1→6)-*O*-[α-l-rhamnopyranosyl-(1→2)]-*O*-β-d-galactopyranoside showed high stability in the N3 binding site. Additionally, Jo *et al.*^36^ reported that herbacetin, rhoifolin, and pectolinarin were found to efficiently block the enzymatic activity of SARS-CoV 3C like protease (3CL^pro^). Moreover, kaempferol, quercetin, and rutin were also able to bind at the substrate-binding pocket of 3CL^pro^ with high affinity (105–106 M^−1^). Additionally, they interact with the active site residues such as His41 and Cys145 through hydrogen bonding and hydrophobic interactions.^37^ These results suggest the potential effect of flavonoids as novel inhibitors of SARS-CoV-2 with comparable potency as that of darunavir.

## 3. Experimental

### 3.1 Plant material

Leaves and bark of *Manilkara hexandra* (Roxb.) Dubard (family: Sapotaceae) was collected in October plants cultivated in the Zoo. The plant was identified by Mrs Terase Labib, Taxonomist of Orman Garden, Giza, Egypt. The identity of the plant was kindly confirmed by Dr Mohamed El-Gebaly, Lecturer of Plant Taxonomy, National Research Center, Giza, Egypt. Voucher specimens (code: M-04-2016) are kept in the Department of Pharmacognosy, Faculty of Pharmacy (Girls), Al-Azhar University.

### 3.2 Extraction and purification of the plant material

The air-dried powdered leaves of *Manilkara hexandra* (Roxb.) Dubard (3 kg) were extracted with hot 70% aqueous MeOH under reflux (3 kg, 5 × 6 L) refluxed for 72 hours at 70 °C then evaporation of MeOH under vacuum to get (521 g) dry extract. The concentrated methanolic extract (521 g) was suspended in 100 mL distilled water and successively extracted with methylene chloride (3 × 750 mL) under reflux for 4 hours on a water bath at 40 °C then separation of the organic layer with the separating funnel and evaporation to obtain (5 g), re-evapourate the aqueous residue *via* a rotary evaporator till dryness and extraction by refluxing with ethyl acetate for 12 hours (4 × 750 mL) on a water bath at 70 °C (10 g), then complete evaporation of the remaining aqueous part, followed by fractionation with butanol by refluxing on a water bath for 36 hours at 70 °C (3 × 1000 mL) which give (12 g), finally re-evaporation of the remaining aqueous part *via* rotary evaporator and extraction with 100% methanol to precipitate sugars and inorganic salts by refluxing on a water bath for 48 hours at 70 °C (6 × 1000 mL) to obtain (210 g). The methanolic extract (210 g) is then applied on polyamide column chromatography using H_2_O then H_2_O/MeOH mixture up to 100% MeOH by using paper chromatography, UV-light, and spray reagents similar fractions of polyamide columns were collected (collected fraction from 1–3). Fractions 1 and 2 were eluted at 20% aqueous methanol then concentrated under vacuum and the residue of collected fraction 1 was subjected to Sephadex LH-20 column chromatography using (butanol : isopropyl alcohol : water) (4 : 1 : 5) as a solvent system then the eluted fraction (6–8) are applied on cellulose column chromatography using gradient elution from 30% aqueous methanol (compound III). While collected fraction 2 residue was subjected to Sephadex LH-20 column chromatography using gradient elution started with 15% aqueous methanol till 100% methanol to obtain compound IV which was eluted at 30% aqueous methanol. Collected fraction 3 eluted with 30% methanol from polyamide column was then submitted to gel filtration on Sephadex LH-20 column chromatography using 100% methanol to obtain compound II. Compound I was eluted at 40% aqueous methanol from the polyamide column. Then absolute ethanol on Sephadex LH-20 was used for purification of the isolated compounds I–IV. Sub-fractions were collected based on comparing paper chromatography and TLC and visualized with UV and sprayed with different reagents.

### 3.3 Metabolomic analysis procedure

Air-dried and finely powdered *Manilkara hexandra* (Roxb.) Dubard leaves and bark (2 g each). Leaves were exhaustively extracted with 70% aqueous methanol (3 × 5 ml) at room temperature and concentrated under vacuum at 40 °C to afford 80 mg crude methanolic extract, while the bark was extracted first with 70% aqueous methanol followed by fractionation with ethyl acetate (3 × 5 mL) at room temperature and concentrated under vacuum at 40 °C to afford 50 mg ethyl acetate extract. The crude extracts were subjected to metabolomic analysis using analytical techniques of LC-HRESIMS.^38–40^ LC-HRESIMS metabolomics analyses were performed on an Acquity Ultra Performance Liquid Chromatography system coupled to a Synapt G2 HDMS quadrupole time-of-flight hybrid mass spectrometer (Waters, Milford, MA, USA). Chromatographic separation was carried out on a BEH C18 column (2.1 × 100 mm, 1.7 μm particle size; Waters, Milford, MA, USA) with a guard column (2.1 × 5 mm, 1.7 μm particle size) and a linear binary solvent gradient of 0–100% eluent B, over 6 min, at a flow rate of 0.3 mL min^−1^, using 0.1% formic acid in water (v/v) as solvent A and acetonitrile as solvent B. The injection volume was 2 μL and the column temperature was 40 °C. After chromatographic separation, the metabolites were detected by mass spectrometry using electrospray ionization (ESI) in the positive mode; the source was operated at 120 °C. The ESI capillary voltage was set to 0.8 kV, the sampling cone voltage was set to 25 V and nitrogen (at 350 °C, a flow rate of 800 L h^−1^) was used as the desolvation gas and the cone gas (flow rate of 30 L h^−1^). The mass range for TOF-MS was set from *m*/*z* (mass-to-charge ratio) 50–1200. In MZmine 2.12, the raw data were imported by selecting the ProteoWizard converted positive files in the mzML format. Mass ion peaks were detected and followed by a chromatogram builder and a chromatogram deconvolution. The local minimum search algorithm was applied, and isotopes were also identified *via* the isotopic peaks grouper. Missing peaks were detected using the gap-filling peak finder. An adduct search, as well as a complex search, was performed. The processed data set was then subjected to molecular formula prediction and peak identification. The positive and negative ionization mode structures from each of the respective plant extracts were dereplicated against the DNP database Dictionary of Natural Products (DNP) database.

### 3.4 Anti-SARS-CoV-2 docking studies

#### 3.4.1 Target compounds optimization

The target compounds were constructed into a 3D model by Molecular Operating Environment® 2019 program. After checking their structures and the formal charges on atoms by the 2D depiction, the following steps were carried out: the target compounds were subjected to a conformational search. All conformers were subjected to energy minimization, all the minimizations were performed until an RMSD gradient of 0.01 kcal mol^−1^ and RMS distance of 0.1 Å with MMFF94X force-field and the partial charges were automatically calculated. The obtained database was then saved as an MDB file to be used in the docking calculations.^41^

#### 3.4.2 Optimization of the enzymes active site

The X-ray crystallographic structure of M^pro^ complexed with N3 was obtained from the Protein Data Bank through the internet (http://www.rcsb.org/pdb/, code 6LU7).^7^ Molecular Operating Environment® 2019 program was used for the preparation of the enzyme for the docking studies by adding hydrogen atoms to the system with their standard geometry. The atoms' connection and type were checked for any errors with automatic correction. The selection of the receptor and its atoms potential were fixed. Site Finder was used for the active site search in the enzyme structure using all default items. Dummy atoms were created from the site finder of the pocket. The docking was validated by the redocking of the energetically minimized ligand N3 and the root mean square deviation that obtained was 1.4228 Å^2^.

#### 3.4.3 Docking of the target molecules to viral main protease binding site

Docking of the conformation database of the target compounds was done after the removal of the ligand N3. The following methodology was generally applied: the enzyme active site file was loaded, and the docking tool was initiated. The program specifications were adjusted to dummy atoms as the docking site, alpha triangle as the placement methodology to be used. The scoring methodology London dG is used and was adjusted to its default values. The MDB file of the ligand to be docked was loaded and dock calculations were run automatically. The poses that showed best ligand–enzyme interactions were selected and stored for energy calculations.^42^

## 4. Conclusion

This study demonstrated the high efficiency of LC-HRESIMS in metabolomic profiling of *Manilkara hexandra* (Roxb.) Dubard different leaves and bark, which dereplicated different chemical metabolomic compounds belonging to different classes, including phenolic, flavones, flavonol glycosides, and triterpenes. A phytochemical investigation of the *Manilkara hexandra* (Roxb.) Dubard leaves afforded isolation of four flavonols for the first time from *Manilkara hexandra* (Roxb.) Dubard and one of them are isolated for the first time from the *Manilkara* genus, which identified as mearnsetin-3-*O*-β-d-rutinoside. Investigating the anti-SARS-CoV-2 activities of compounds (1–11) using molecular docking simulation study showed that rutin, myricitrin, mearnsitrin, and quercetin 3-*O*-β-d-glucoside are promising SARS-CoV-2 main protease inhibitors. The study high lights that rutin which is a citrus flavonoid glycoside and can be found in green tea, grape seeds, red pepper, apple, citrus fruits, berries, and peaches^34^ have the highest activity as SARS-CoV-2 protease inhibitor followed by quercetin 3-*O*-β-d-glucoside, mearnsitrin, and myricitrin which can be found in vegetables, fruits, nuts, and spices.^43^ It worth mentioning that rutin has a very impressive pharmacological profile and can possess different therapeutic activities *e.g.* anti-inflammatory and antiviral activity.^44^ So, based on the above information, there is an urgent need for *in vivo* studies of the anti-SARS-CoV-2 potential of rutin which will give a hopeful and safe insight in discovering a targeting drug against the current SARS-CoV-2 pandemic.

## Conflicts of interest

We declare that we have no conflict of interest.

## Supplementary Material

RA-010-D0RA05679K-s001
