# Supplementary material for: Inhibition of SARS-CoV-2 main protease by phenolic compounds from Manilkara hexandra (Roxb.) Dubard assisted by metabolite profiling and in silico virtual screening
Source: RSC Adv. 2020 Aug 28;10(53):32148–55. doi: 10.1039/d0ra05679k (PMC9056568; doi:10.1039/d0ra05679k)
Supplement: RA-010-D0RA05679K-s001 [file RA-010-D0RA05679K-s001.pdf]

Electronic Supplementary Material (ESI) for RSC Advances.
This journal is © The Royal Society of Chemistry 2020

Inhibition of SARS-CoV-2 main protease by phenolic compounds from *Manilkara hexandra* (Roxb.) Dubard assisted by *in Silico* virtual screening

Fatma M. Abd El-Mordy[1], Mohamed M. El-Hamouly[2], Magda T. Ibrahim[3], Gehad Abd El-Rheem[4], Omar M. Aly[5], Adel M. Abd El-kader[6,7], Khayrya A. Youssif[8], Usama Ramadan Abdelmohsen[6,9]*

[1]Department of Pharmacognosy, Faculty of Pharmacy (Girls), Al-Azhar University, 11754 Cairo, Egypt.

[2]Department of Pharmacognosy, Faculty of Pharmacy (Boys), Al-Azhar University, 11371 Cairo, Egypt.

[3]Department of Pharmacognosy, Faculty of Pharmacy, Sinai University, 41636 Kantara Branch.

[4]Department of Pharmacology, National Research Centre, 12622 Giza, Egypt.

[5]Department of Medicinal Chemistry, Faculty of Pharmacy, Minia University, 61519 Minia, Egypt.

[6]Department of Pharmacognosy, Faculty of Pharmacy, Deraya University, 61111 Minia, Egypt

[7]Department of Pharmacognosy, Faculty of Pharmacy, Al-Azhar University, Assiut 71524, Egypt

[8]Department of Pharmacognosy, Faculty of Pharmacy, Modern University for Technology and Information, Cairo, Egypt.

[9]Department of Pharmacognosy, Faculty of Pharmacy, Minia University, 61519 Minia, Egypt.

***Corresponding author: Usama.ramadan@mu.edu.eg**

Table of Contents

**Figure S1:**	UV spectra of Compound **I**.	**7**
**Figure S2:**	^{1}H-NMR spectrum of compound **I** in (DMSO-d_6- 400 MHz).	**8**
**Figure S3:**	^{13}C-NMR spectrum of compound **I** in (DMSO-d_6- 100 MHz).	**9**
**Figure S4:**	UV spectra of compound **II**.	**10**
**Figure S5:**	Negative ESI/MS spectra of compound **II**.	**11**
**Figure S6:**	^{1}H-NMR spectrum of compound **II** in (DMSO-d_6- 400 MHz).	**12**
**Figure S7:**	^{13}C-NMR spectrum of compound **II** in (DMSO-d_6- 100 MHz).	**13**
**Figure S8:**	Partial expansion of the ^{13}C-NMR spectrum of compound **II** in (DMSO-d_6- 100 MHz).	**14**
**Figure S9:**	^{1}H-NMR spectrum of compound **III** in (DMSO-d_6- 400 MHz).	**15**
**Figure S10:**	Partial expansion of the ^{1}H-NMR spectrum of compound **III** in (DMSO-d_6- 400 MHz).	**16**
**Figure S11:**	^{13}C-NMR spectrum of compound **III** in (DMSO-d_6- 100 MHz).	**17**
**Figure S12:**	Partial expansion of the ^{13}C-NMR spectrum of compound **III** in (DMSO-d_6- 100 MHz).	**18**
**Figure S13:**	DEPT-135 spectrum of compound **III** in (DMSO-d_6- 100 MHz).	**19**
**Figure S14:**	^{1}H-NMR spectrum of compound **IV** in (DMSO-d_6- 400 MHz)	**20**
**Figure S15:**	Partial expansion of the ^{1}H-NMR spectrum of compound **IV** in (DMSO-d_6- 400 MHz).	**21**
**Figure S16:**	DEPT-135 spectrum of compound **IV** in (DMSO-d_6- 100 MHz)	**22**
**Figure S17:**	Partial expansion of the DEPT-135 spectrum of compound **IV** in (DMSO-d_6- 100 MHz).	**23**
**Figure S18:**	Metabolomic chromatogram of different extracts of *Manilkara hexandra* (Roxb.) Dubard.	**24**
**Figure S19:**	Metabolomic chromatogram of the ethyl acetate extract of *Manilkara hexandra* (Roxb.) Dubard bark.	**25**
**Figure S20:**	Metabolomic chromatogram of the methanol extract of *Manilkara hexandra* (Roxb.) Dubard leaves.	**26**

Methods:

Compound I

UV/Vis λ_{max} (MeOH) *nm*: 254, 302, 377; (+ NaOMe): 284 sh, 317, 416; (+ NaOAc): 265, 332, 385; (+ NaOAc/H_3BO_3): 232.5, 258, 300; (+ $AlCl_3$): 269, 309 sh, 447 ;(+ $AlCl_3$/HCl): 269, 314, 428.

Positive MS, m/z 319 $[M+H]^+$ for a MF: $C_{15}H_{10}O_8$.

^{1}H NMR (400 MHz, DEMSO-d_6, TMS as int. std , δ, ppm): 12.69 (br s, l H , 5-OH), 10.91-9.28 (br s , 4H, 7 , 3' , 4' , 5' - OH) 6.89 (s, 2H, H-2' , 6') , 6.39 (d, l H , *J*=2.1 Hz, H-8), 6.21 (d, l H , *J*=2.1 Hz, H - 6), ^{13}C NMR (100 MHz, DEMSO-d_6) 157.96 (C-2), 134.74 (C-3), 178.24 (C-4), 161.76 (C-5), 99.13 (C-6), 164.63 (C-7), 94.00 (C-8), 156.87 (C-9), 104.50 (C-10), 120.08 (C-1'), 108.38 (C-2`, C-6'), 146.22 (C-3', C-5'), 136.91 (C-4').

Compound II

UV/Vis λ_{max} (MeOH) *nm*: 257.5, 301.5, 352.5; (+ NaOMe): 270.5 sh, 322, 391.5; (+ NaOAc): 271, 322.5, 381; (+ NaOAc/H_3BO_3): 265, 339, 376.5; (+ $AlCl_3$): 273, 312 sh, 365.5, 420 ;(+ $AlCl_3$/HCl): 272.5, 302.5, 369.5, 422.5.

Negative MS, m/z 463.0 $[M-H]^-$ for a MF: C_{21} $H_{19}O_{12}$, 316.04 $[M\text{- rhamnose}]^-$.

^{1}H NMR (400 MHz, DEMSO-d_6, TMS as int. std , δ, ppm): 12.69 (br s, l H , 5-OH), 10.86-9.27 (br s , 4H, 7 , 3 ' , 4 ' , 5 ' - OH), 6.90 (s, 2H, H-2' , 6') , 6.38 (d, l H , *J*=2 Hz, H-8), 6.21 (d, l H , *J*=2 Hz, H - 6) , 5.21 (br *s,* lH, H-1" of rhamnose), 3.99-3.17 (m, sugar protons) , 0.86 (d, *J*= 6 Hz, 3H, rhamnose - CH_3), ^{13}C NMR (100 MHz, DEMSO-d_6) 157.96 (C-2), 134.74 (C-3), 178.24 (C-4), 161.77 (C-5), 99.14 (C-6), 164.63 (C-7), 94.00 (C-8), 156.87 (C-9), 104.51 (C-10), 120.09 (C-1`), 108.38 (C-2`, C-6`), 146.22 (C-3`, C-5`), 136.91 (C-4`), 102.37 (C-1``), 70.85 (C-2``), 71.02 (C-3``), 71.73 (C-4``), 70.48 (C-5``), 17.99 (C-6``).

Compound III

UV/Vis λ_{max} (MeOH) *nm*: 265, 298sh, 338sh; (+ NaOMe): 271, 328sh, 372; (+ NaOAc): 272, 340sh; (+ NaOAc/H_3BO_3): 265, 305, 340sh; (+ $AlCl_3$): 274, 302, 340, 392sh; (+ $AlCl_3$/HCl): 274, 302, 340, 392sh.

Positive MS, m/z 479.0 $[M_+H]^+$ for a MF: $C_{22}H_{22}O_{12}$, 333 $[\text{aglycon}_+H]^+$.

^{1}H NMR (400 MHz, DEMSO-d_6, TMS as int. std , δ, ppm) 12.58 (br s, l H , 5-OH), 10.95-9.47 (br s , 3H, 7 , 3 ' , 5 ' - OH) 6.82 (s, 2H, H-2' , 6 ') , 6.39 (d, l H , *J*=2.1 Hz, H-8), 6.22 (d, l H , *J*=2.1 Hz, H - 6) , 5.16 (br s, lH, H-1" of rhnmnose), 3.75 (s , 3H, 4'-OCH_3), 3.99-3.15 (m, sugar protons) , 0.83 (d, *J*=6 Hz, 3H, rhamnose - CH_3),

^{13}C NMR (100 MHz, DEMSO-d_6) 157.91 (C-2), 135.17 (C-3), 178.04 (C-4), 161.36 (C-5), 99.30 (C-6), 164.44 (C-7), 94.33 (C-8), 156.94 (C-9), 104.67 (C-10), 125.034 (C-1\`), 108.83 (C-2\`, C-6\`), 150.73 (C-3\`, C-5\`), 138.20 (C-4\`), 102.28 (C-1\`\`), 70.33 (C-2\`\`), 70.96 (C-3\`\`), 71.40 (C-4\`\`), 70.52 (C-5\`\`), 17.42 (C-6\`\`), 60.45 (C4'-O-CH_3).

Compound IV

Positive MS, m/z, 641 $[M_+ H]^+$ for a MF: $C_{28}H_{32}O_{17}$.

^1H NMR (400 MHz, DEMSO-d_6, TMS as int. std, δ, ppm): 12.58 (br s, l H , 5-OH), 10.89-9.47 (br s , 3H, 7 , 3 ' , 5 ' - OH) 6.82 (s, 2H, H-2' , 6 ') , 6.38 (d, l H , *J*=2 Hz, H-8), 6.22 (d, l H , *J*=2 Hz, H - 6) , 5.16 (br s, lH, H-1''' of rhnmnose), 4.95 (d, 1H,*J*= 6.6 Hz, H-1'' of glucose), 3.75 (s , 3H, 4'-OCH_3), 4.15-3.15 (m, sugar protons) , 0.82 (d, *J*=5.8 Hz, 3H, rhamnose - CH_3). ^{13}C NMR (100 MHz, DEMSO-d_6) **(Table 2)** 157.91 (C-2), 135.17 (C-3), 178.04 (C-4), 161.36 (C-5), 99.25 (C-6), 164.44 (C-7), 94.12 (C-8), 156.94 (C-9), 104.67 (C-10), 129.12 (C-1\`), 109.09 (C-2\`, C-6\`), 150.73 (C-3\`, C-5\`), 138.20 (C-4\`), 102.63 (C-1\`\`), 73.64 (C-2\`\`), 77.13 (C-3\`\`), 70.51 (C-4\`\`), 77.50 (C-5\`\`), 67.87 (C-6\`\`), 101.23 (C-1\`\`\`), 70.80 (C-2\`\`\`), 71.01 (C-3\`\`\`), 71.62 (C-4\`\`\`), 69.87 (C-5\`\`\`), 17.93 (C-6\`\`\`), 60.24 (C4'-O-CH_3).

Results and discussion:

Compound I was obtained as a yellow amorphous powder (35mg), melting point 357-359 ˚C. It exhibited a yellow fluorescence spot under long UV light turned to yellowish orange with Naturstoff and gave a faint blue color with $FeCl_3$. UV spectrum of compound I (Supplementary Materials, Figure S1) indicates a flavonol nucleus with free OH at 4\`position, the free hydroxyl group at C-3, C-5, C-7, and orthodihydroxy group at 3\` and 4\` position. The ^1H-NMR spectrum (Supplementary Materials, Figure S2) exhibited a characteristic meta-coupled proton signal at δ 6.21 (1H, d, *J* = 1.4 Hz) and 6.38 (1H, d, *J* = 1.4 Hz) corresponding to H-6 and H-8 of flavonoid A ring. The other AX coupling system at δ 6.89 (2H, br s) was assigned to H-2' and H-6' of B ring. The ^{13}C-NMR spectrum of **I** (Supplementary Materials, Figure S3) revealed the presence of 15 carbon signals from which two signals were representing two equivalent carbons δ 146.03 at (C-3\`, 5\`) and δ 108.38 (C-2\`, 6\`) pairs of equivalent carbons. Eight carbon resonances are aromatic oxygenated at δ 164.63 (C-7), 161.76 (C-5), 156.87 (C-9), 157.96 (C-2), 134.79 (C-4'), 134.74(C-3), six aromatic non-oxygenated carbons at δ 120.08 (C-1\`), 108.38(C-2\`/6\`), 104.50 (C-

10), 99.13 (C-6), 94.00 (C-8) and one carbonyl signal at 178.24(C-4). By comparing the NMR spectral data with those reported in the literature, the structure of compound **I** was determined as 3, 5, 7, 3', 4', 5'- hexahydroxy – flavone (Myricetin) [29].

Compound II was obtained as a yellow amorphous powder (578 mg). A deep purple fluorescence spot appeared under long UV light turned to yellowish-orange color with Naturstoff and faint blue color with $FeCl_3$. UV spectrum of compound II (Supplementary Materials, Figure S4) is similar to that of compound **I**. Its molecular formula was established as $C_{21}H_{20}O_{12}$ based on an ion peak $[M-H]^-$ at m/z 463 in -ve ESI/MS (Supplementary Materials, Figure S5). The spectroscopic data of **II** were similar to **I** (Supplementary Materials, Figure S6, S7, S8) except for the appearance of an α-Lrhamnopyranosyl moiety. So, the ^{1}H-NMR spectrum of **II** (Supplementary Materials, Figure S6)showed the presence of an anomeric proton signal at δ 5.21 (1H, br s), a methyl signal at δ 0.86 (3H, d, J = 6 Hz) and of six additional carbon signals at δ 102.37 (C-1''), 70.85 (C-2''), 71.02 (C3''), 71.73 (C-4''), 70.48 (C-5''), and 17.99 (C-6''). From these data together with ^{13}C-NMR spectral data(Supplementary Materials, Figure S7, S8) indicating compound II was identified as Myricetin 3-*O*-α-L-$^1C_4$ rhamnopyranoside (Myricitrin)[30]

Compound III was obtained as yellow needles (19 mg), on PC. It showed a purple spot under UV and UV/NH_3. Its spectroscopic data were similar to compound **II** (Supplementary Materials, Figure S9-S13). The ^{1}H-NMR spectrum of **III** showed myricetin skeleton in addition to the presence of an anomeric proton signal of rhamnose at δ 5.16 (1H, br s), a methyl signal at δ 0.83 (3H, d, J = 6 Hz) and an additional singlet signal at δ 3.75 (s,3H) indicating 4'-OCH_3 (Supplementary Materials, Figure S9, S10). ^{13}C-NMR spectrum of compound III showed carbon resonances which are characteristic for myricetin aglycone in addition to six carbon signals of rhamnose moiety at δ 102.28 (C-1''), 70.33 (C-2''), 70.96 (C3''), 71.40 (C-4''), 70.52 (C-5''), and 17.42 (C-6'') and an additional signal at δ 60.45 indicating 4'-OCH_3 (Supplementary Materials, Figure S11, S12, S13). From the previous data, compound **III** was identified as Myricetin-4'-*O*-methyl ether-3-*O*-α-L-rhamnopyranoside (**Mearnsitrin)** [19]**.**

Compound IV was obtained as a dark yellow amorphous powder (16.6 mg), on PC. it showed a purple spot under UV and UV/NH_3. *UV/Vis* λ_{max} data is similar to that of compound III. Compound IV gives the typical signals of myricetin aglycon in ^{1}H-

NMR and ^{13}C- NMR (Supplementary Materials, Figure S14-S17), in addition to a single signal in ^{1}H-NMR at 3.75 representing 3H indicating the presence of methoxy group, the OCH3 position is confirmed by ^{13}C- NMR which shows a downfield shift of C-4' to δ 138.20 and a signal at δ 60.24 ppm indicating C-4'-OCH3 (similar to compound **III**). In addition to the presence of the two anomeric protons in the ^{1}H-NMR spectrum (Supplementary Materials, Figure S14-S15) at δ 5.16 (1H, br **s**) and δ 4.95 (**1H,d**, J= 6.6 Hz) together with a signal at 0.82 (**d**, J=5.8 Hz), and two anomeric carbons in the ^{13}C-NMR spectrum(Supplementary Materials, Figure S16-S17) at δ 101.23 and δ 102.63 ppm indicating rhamnose and glucose sugar moiety respectively, the downfield shift of C-6'' of glucose to δ 67.87 indicating rutinoside structure. From these data, compound IV was identified as Mearnsetin-3-*O*-β-D-rutinoside [19].

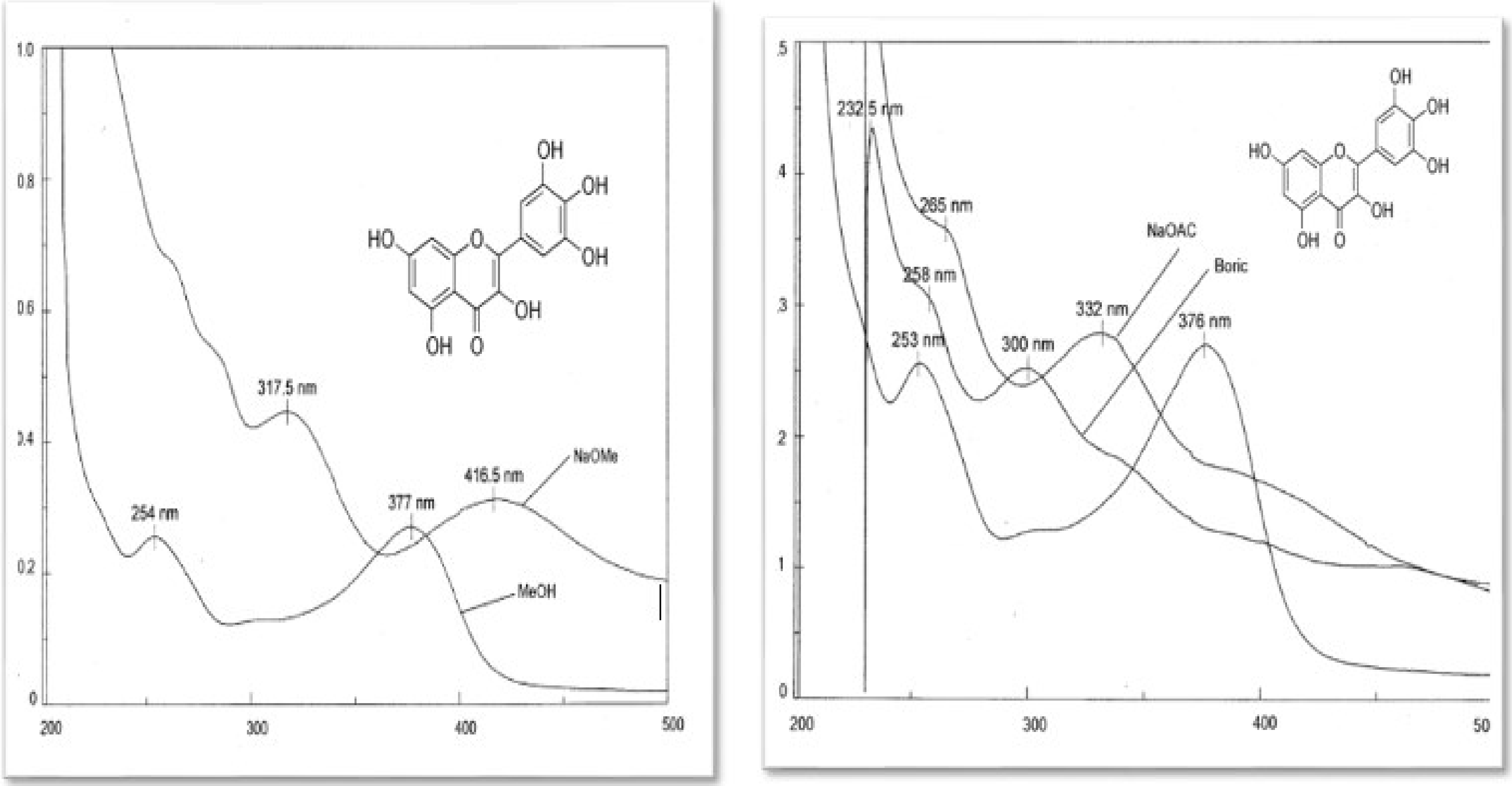

Figure S1: UV spectra of Compound I

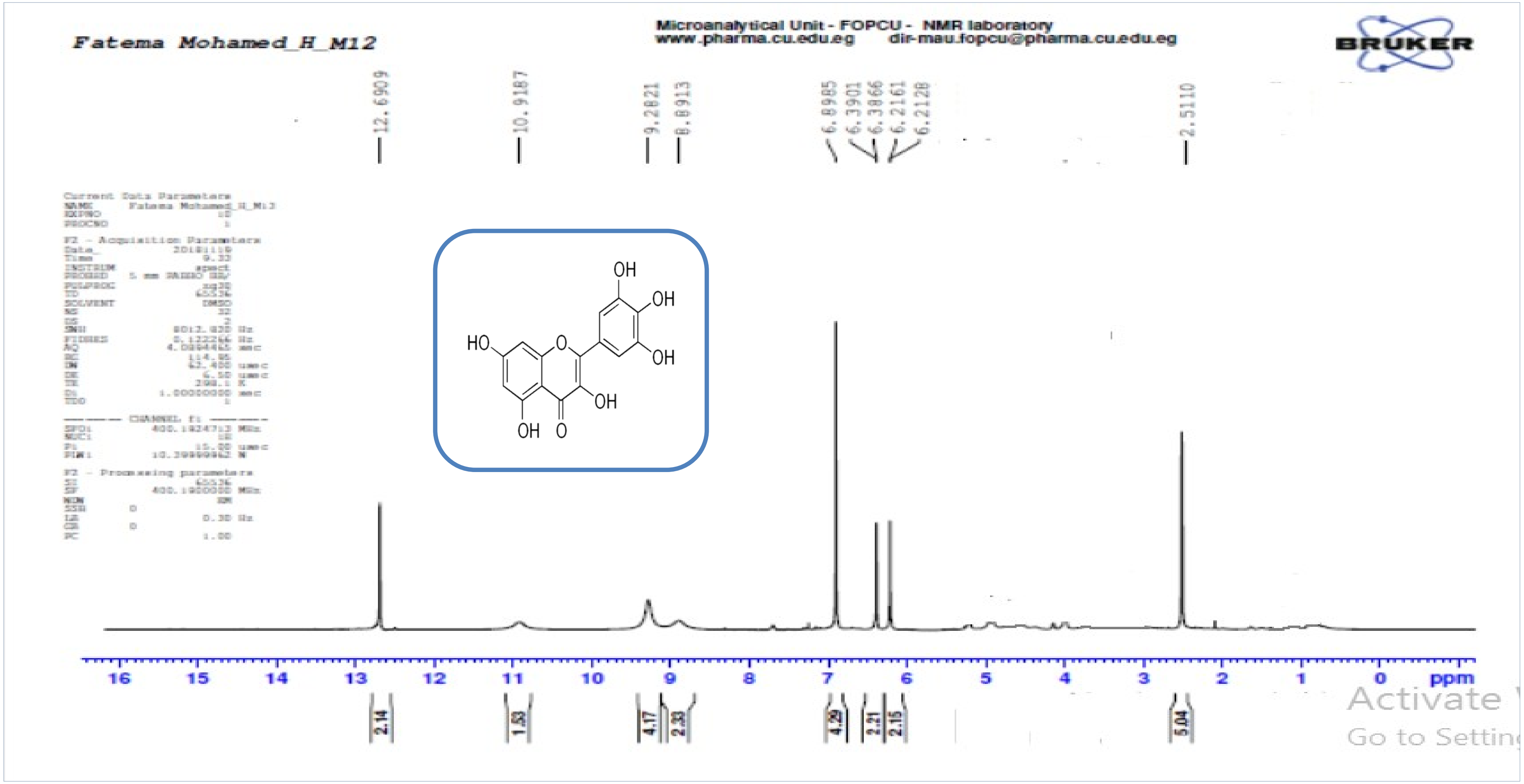

Figure S2: ^{1}H-NMR spectrum of compound I in (DMSO-d_6, 400 MHz)

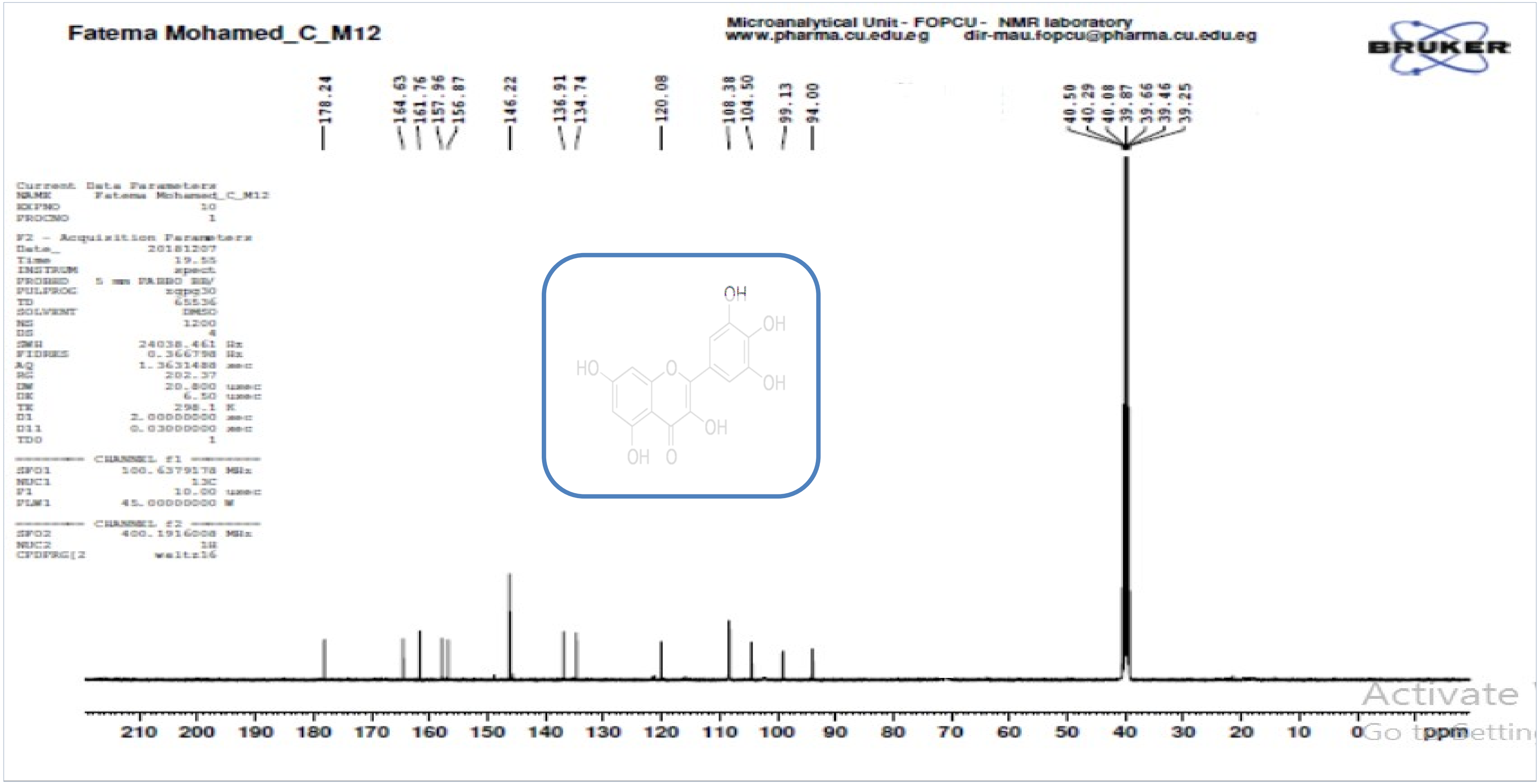

Figure S3: **^{13}C-NMR spectrum of compound I in (DMSO-d_6- 100 MHz)**

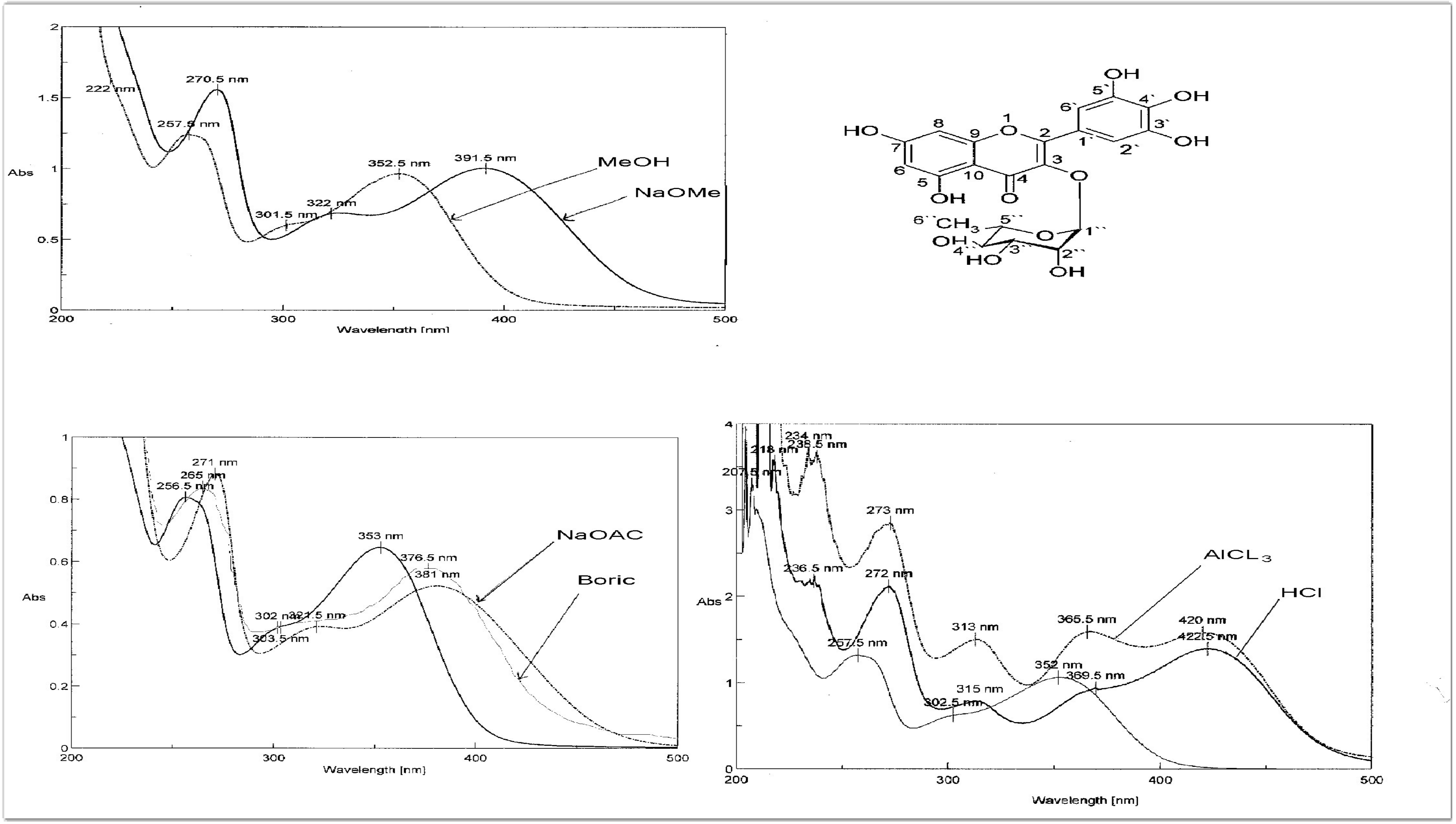

Figure S4: UV spectra of Compound II

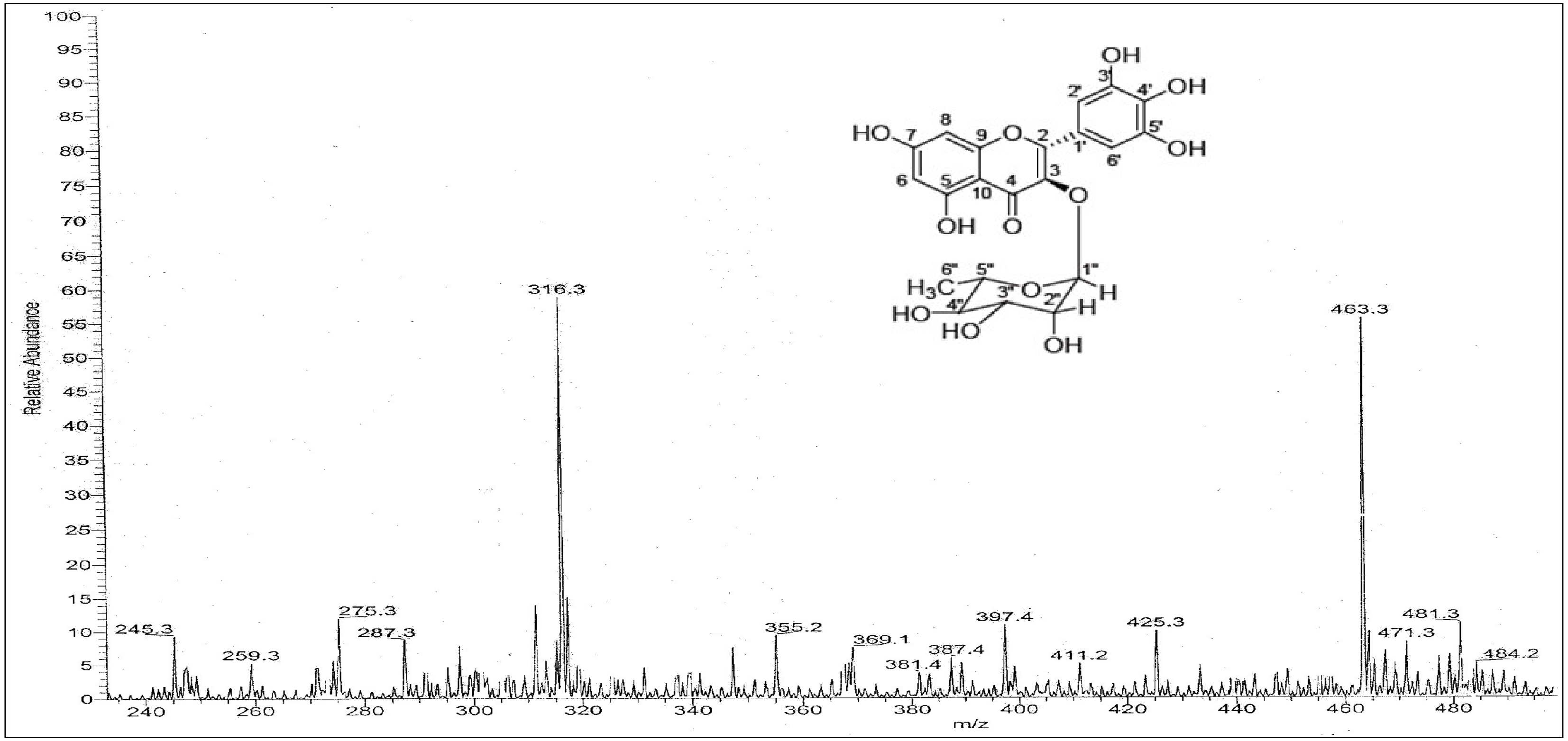

Figure S5: Negative ESI/MS spectra of compound II.

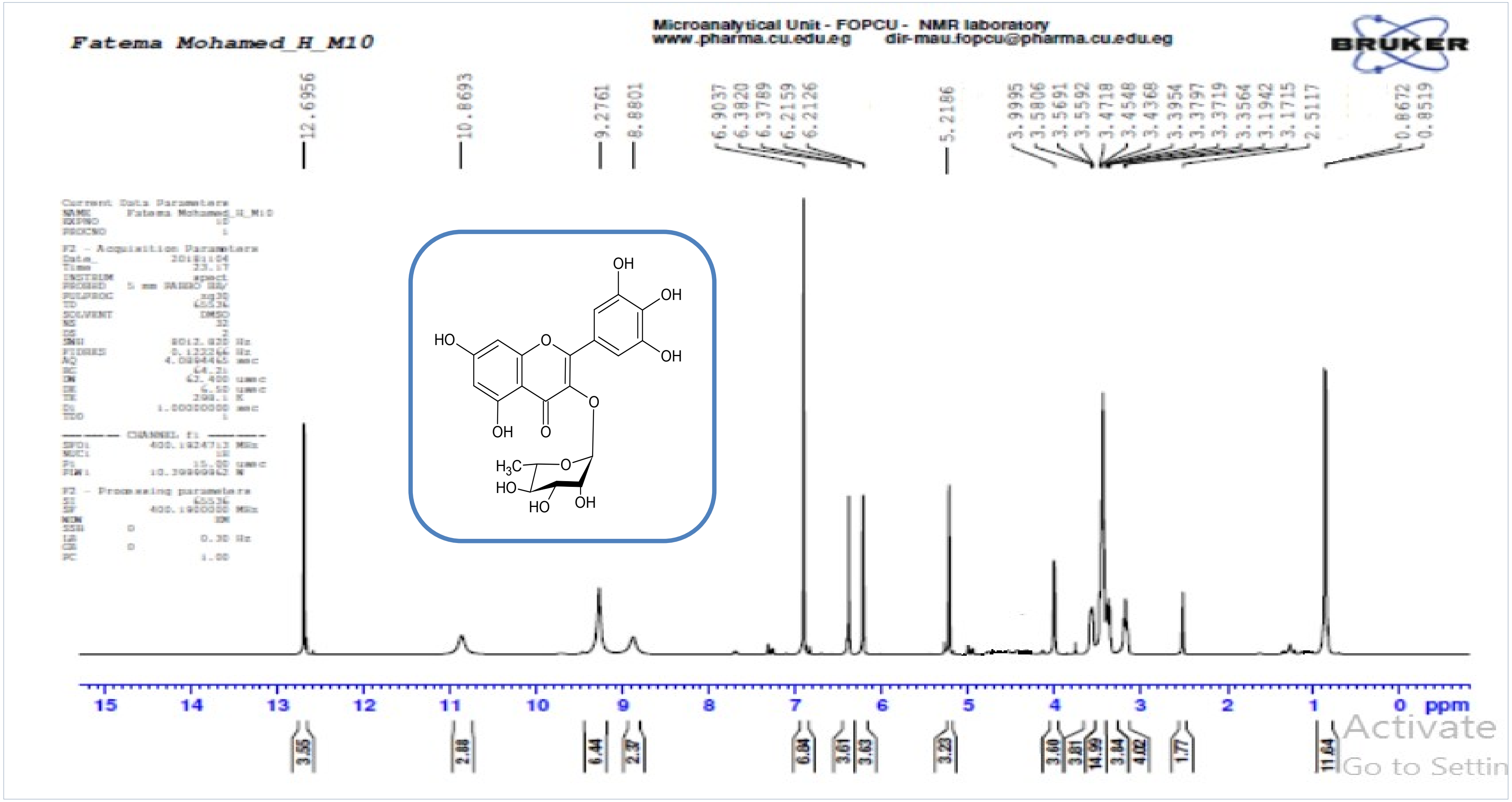

Figure S6: ^{1}H-NMR spectrum of compound II in (DMSO-d_{6}- 400 MHz)

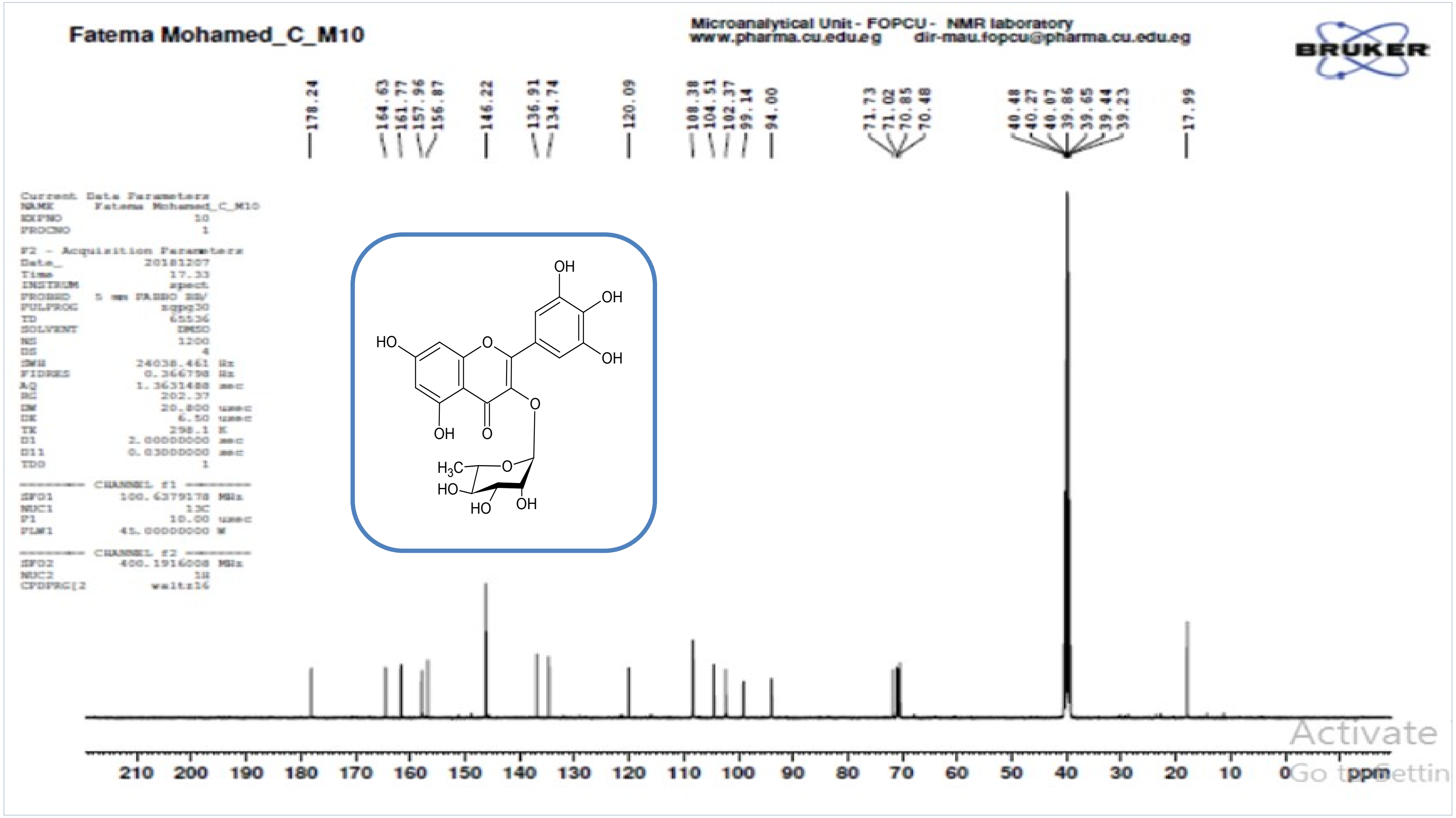

Figur

e S7: ^{13}C-NMR spectrum of compound **II** in (DMSO-d_6. 100 MHz)

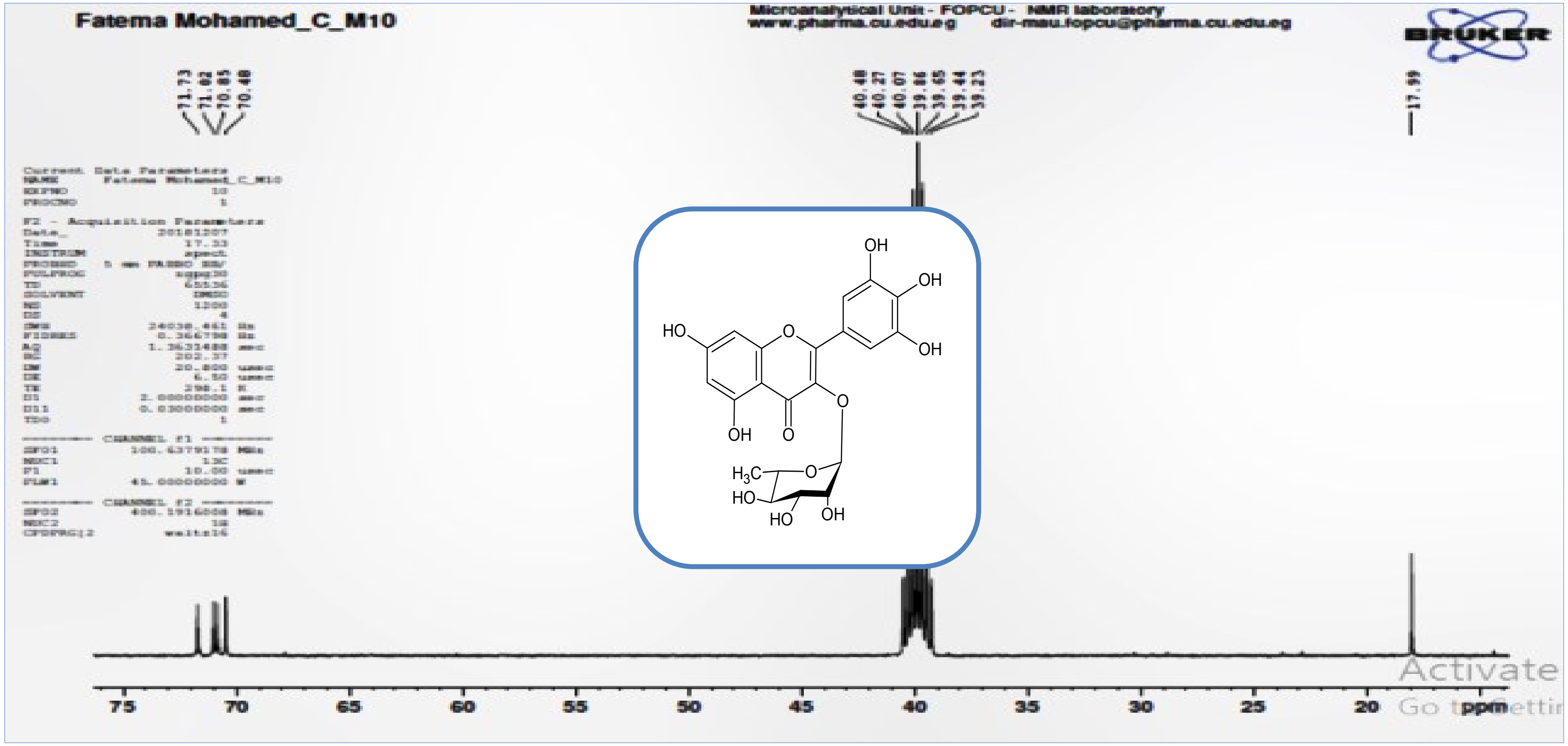

Figure S8: Partial expansion of the ^{13}C-NMR spectrum of compound **II** in (DMSO-d_6, 100 MHz)

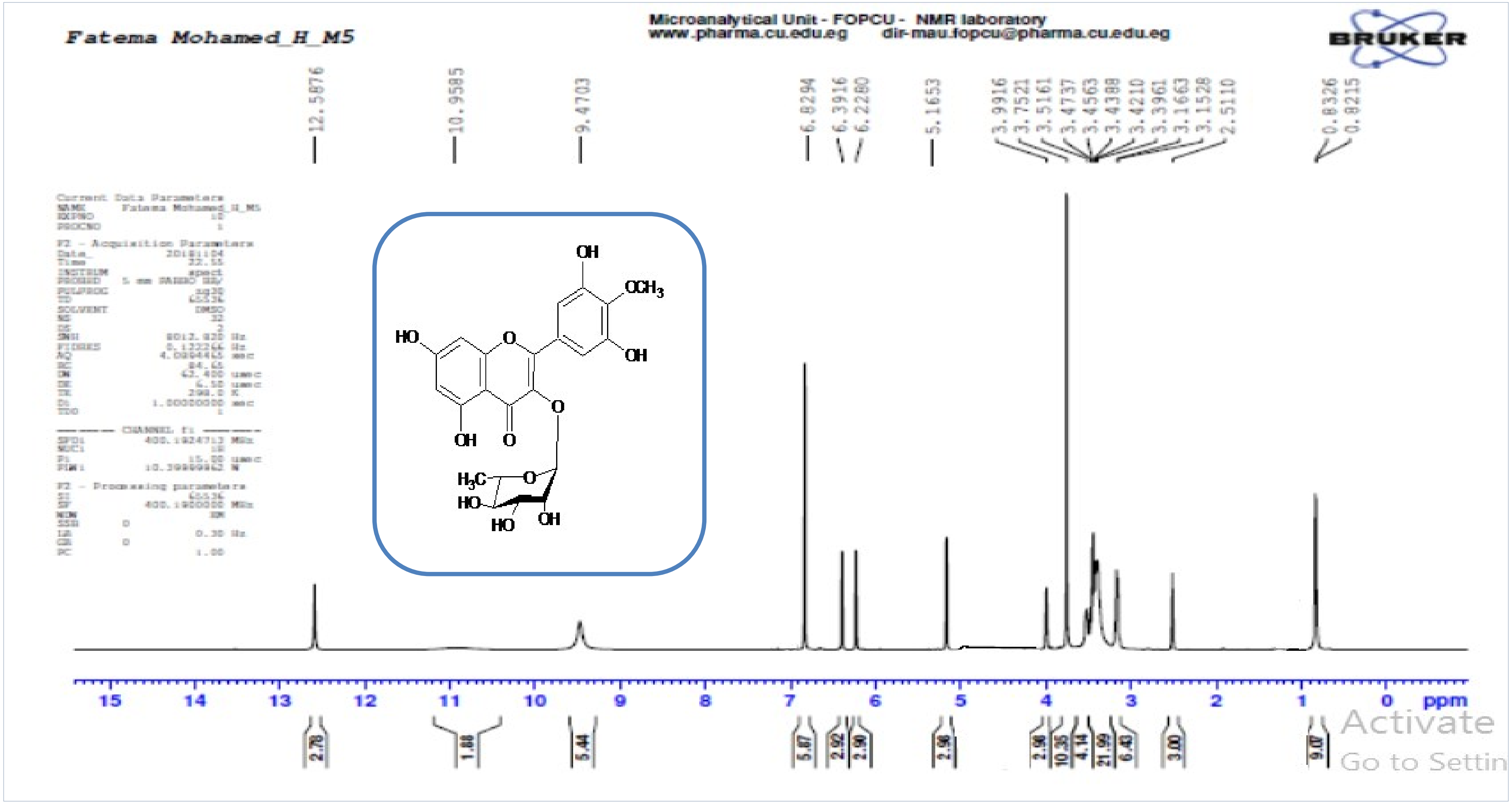

Figure S9: ^{1}H-NMR spectrum of compound **III** in (DMSO-d_6, 400 MHz)

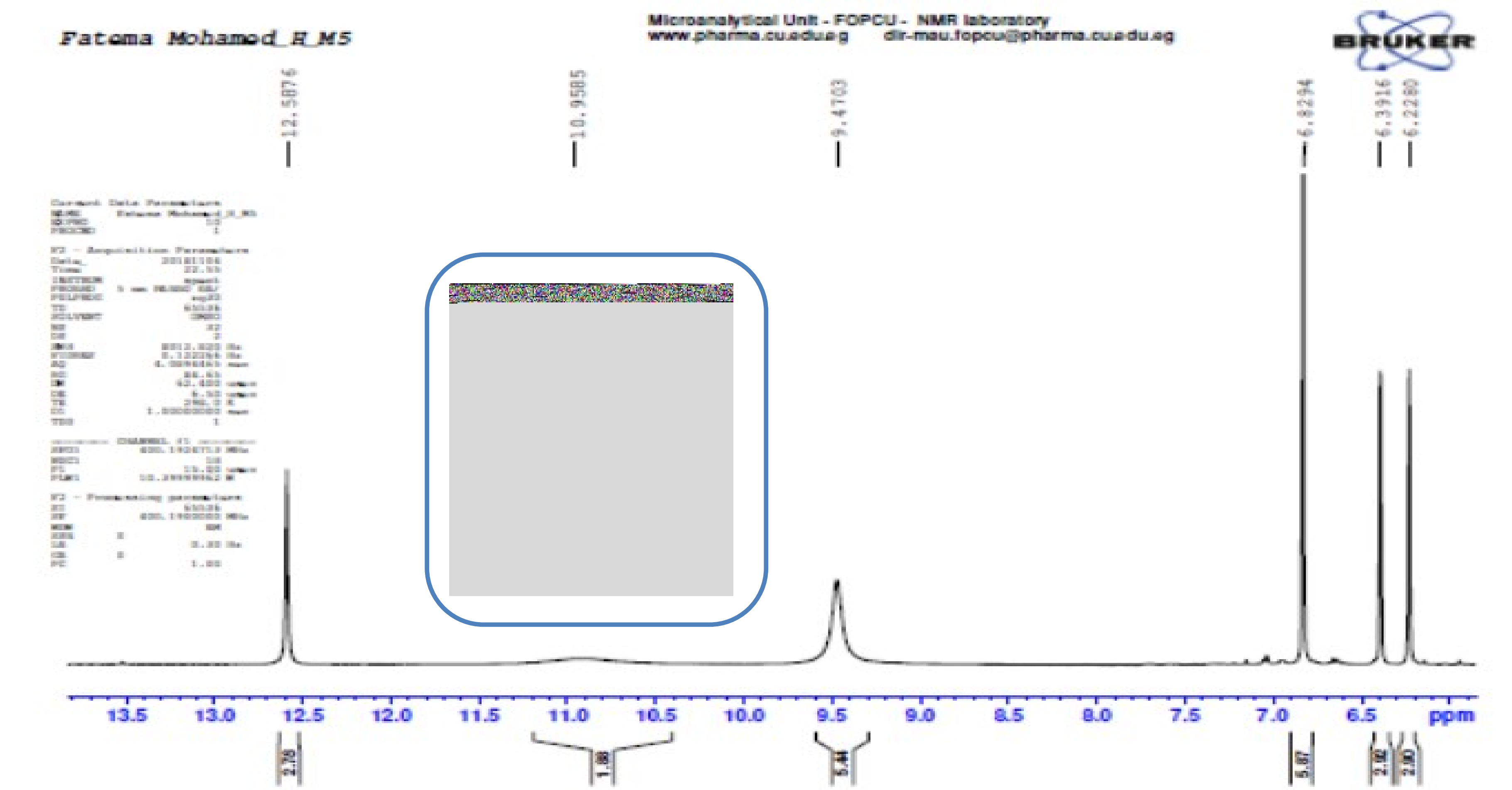

Figure S10: Partial expansion of the ^{1}H-NMR spectrum of compound **III** in (DMSO-d_{6}- 400 MHz)

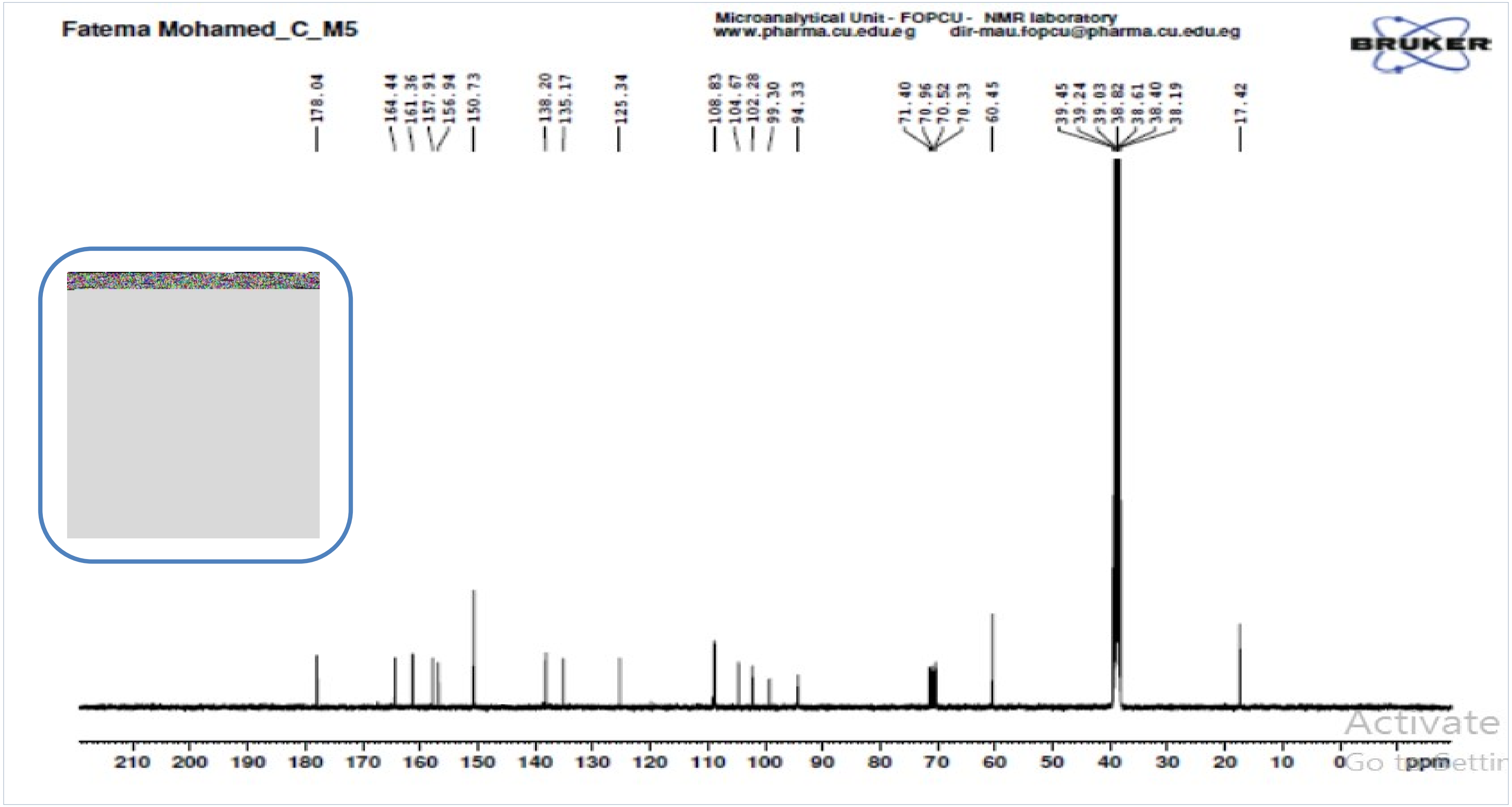

Figure S11: ^{13}C-NMR spectrum of compound **III** in (DMSO-d_6. 100 MHz)

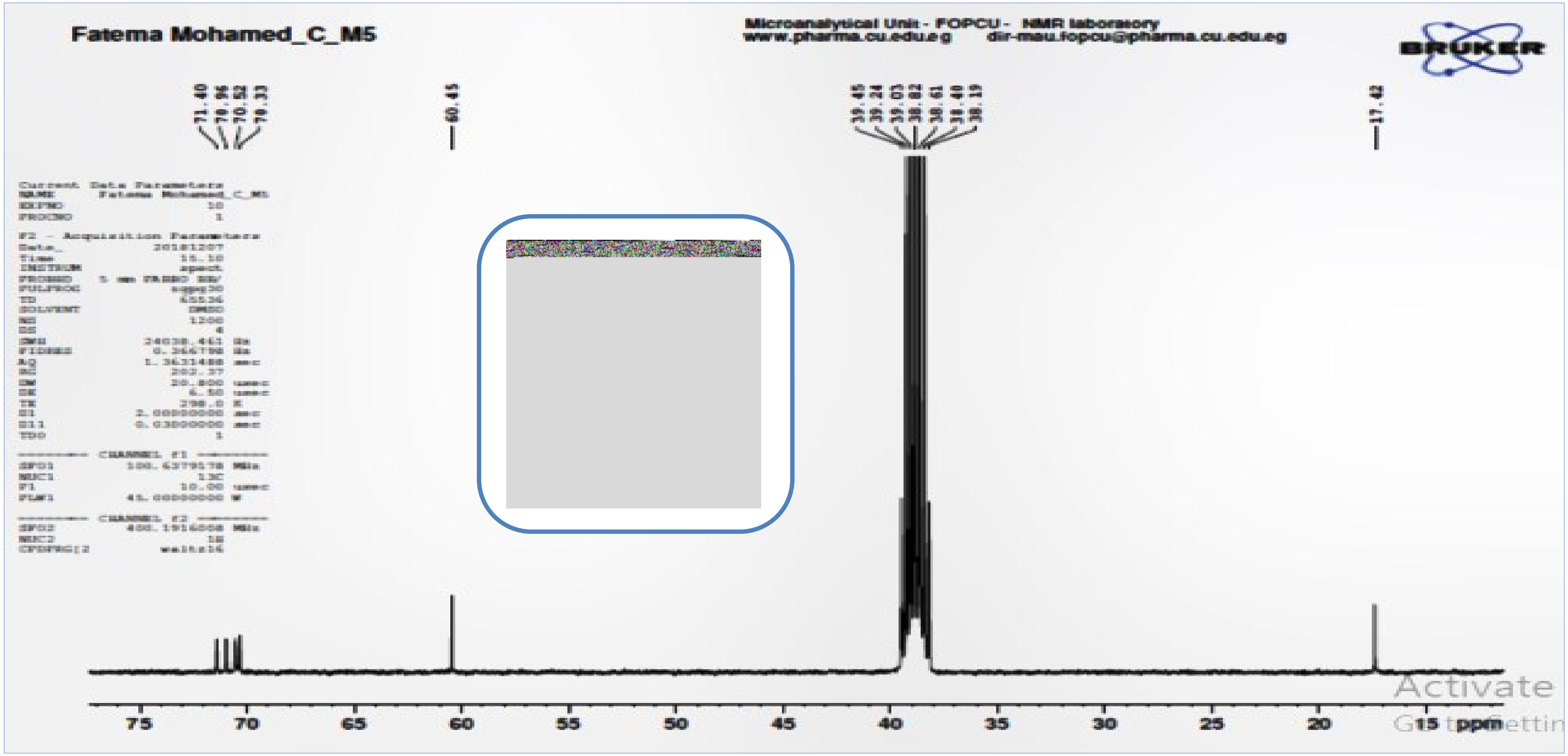

Figure S12: Partial expansion of the ^{13}C-NMR spectrum of compound **III** in (DMSO-d_6- 100 MHz)

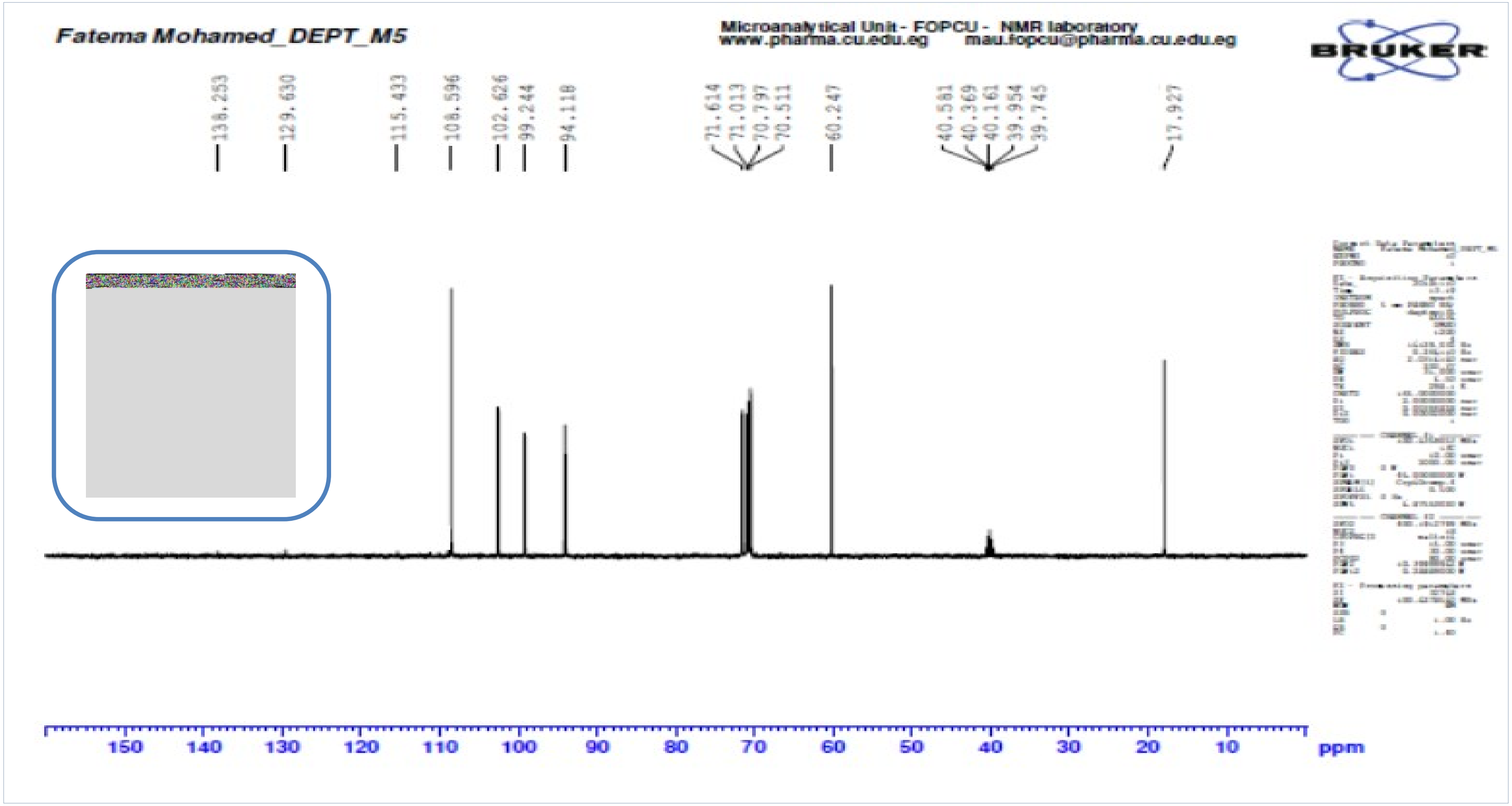

Figure S13: DEPT-135 spectrum of compound **III** in (DMSO-d_6- 100 MHz)

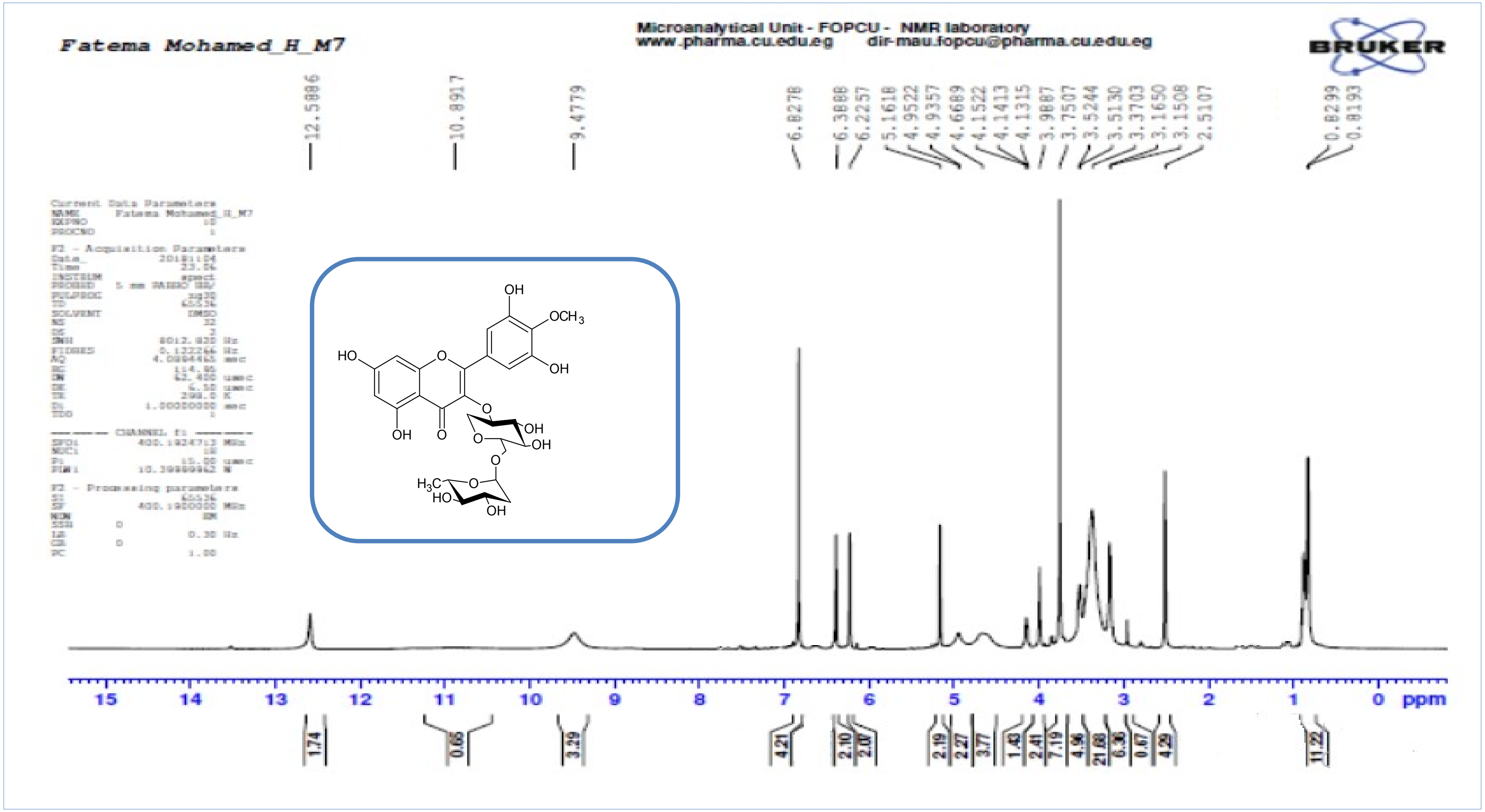

Figure S14: ^{1}H-NMR spectrum of compound **IV** in (DMSO-d_6- 400 MHz)

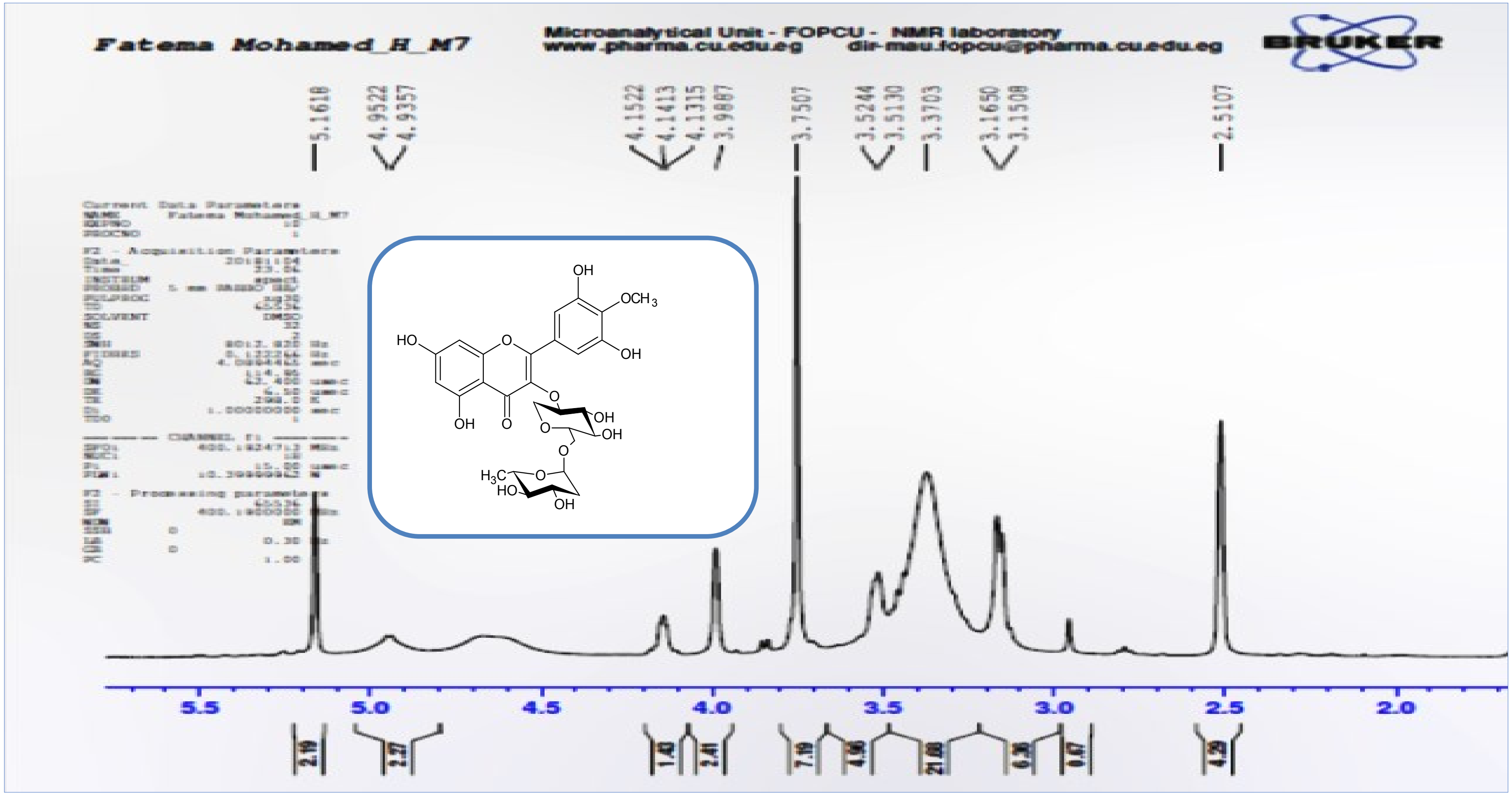

Figure S15: Partial expansion of the ^{1}H-NMR spectrum of compound **IV** in (DMSO-d_6. 400 MHz)

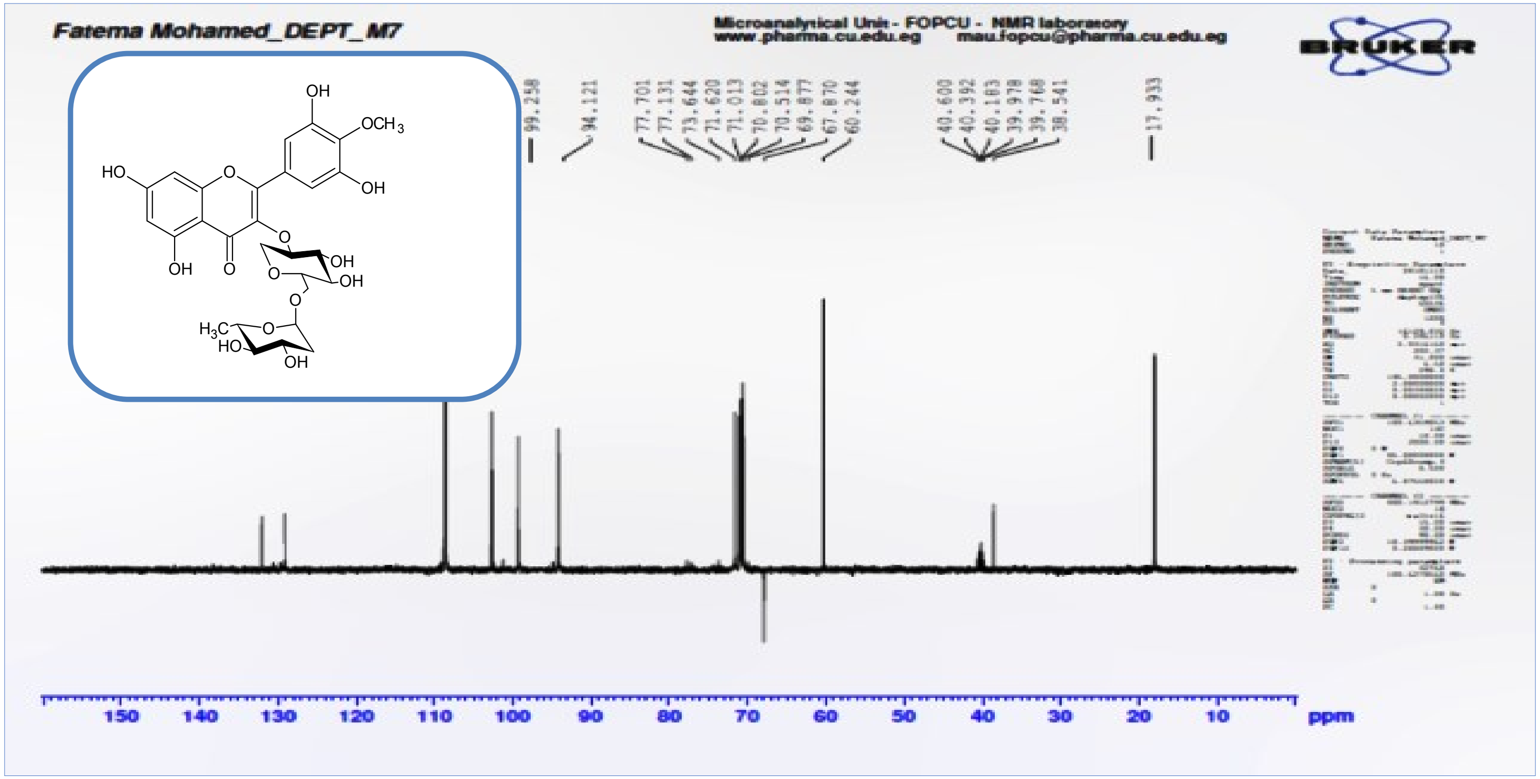

Figure S16: DEPT-135 spectrum of compound **IV** in (DMSO-d_6- 100 MHz)

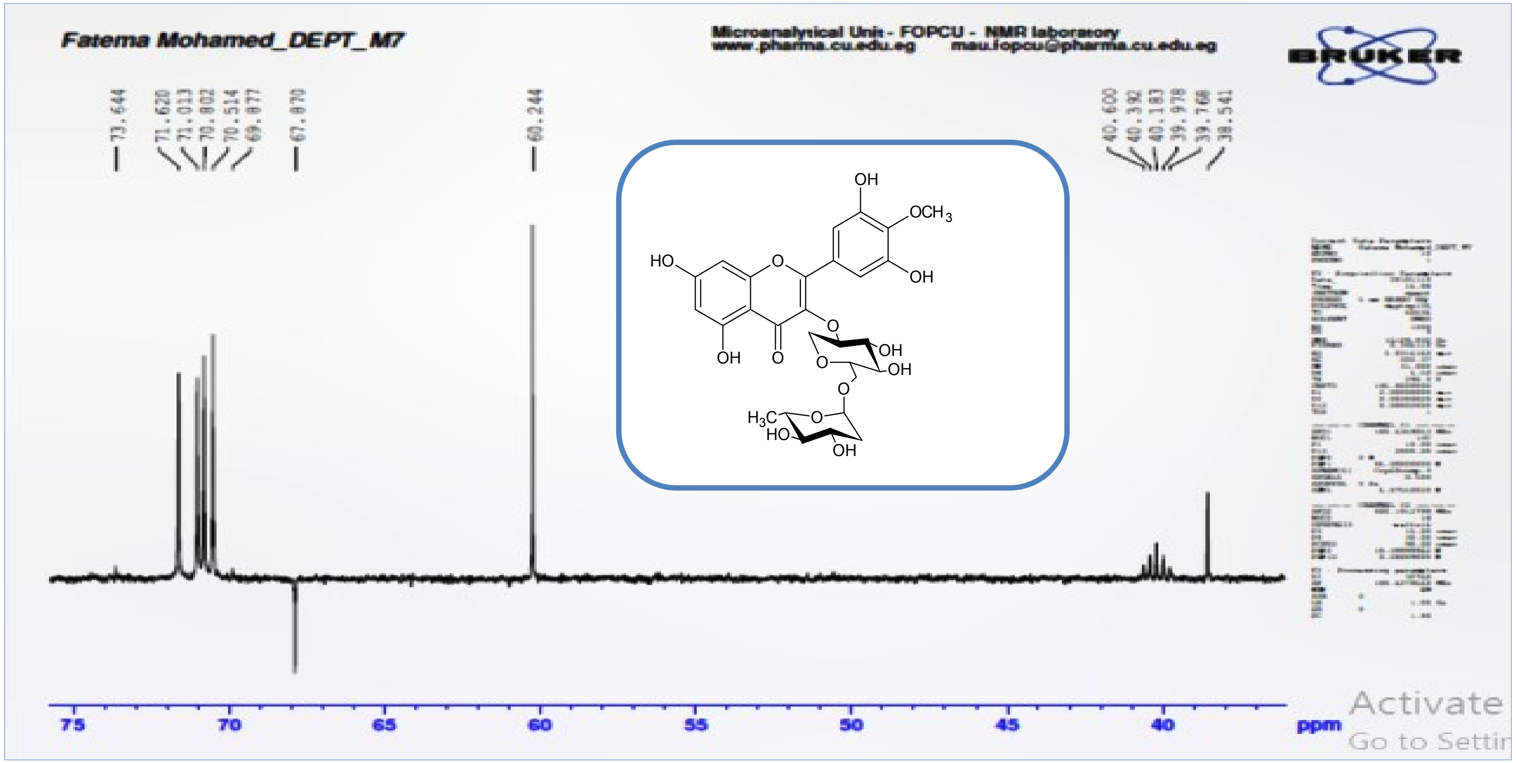

Figure S17: Partial expansion of the DEPT-135 spectrum of compound **IV** in (DMSO-d_6, 100 MHz)

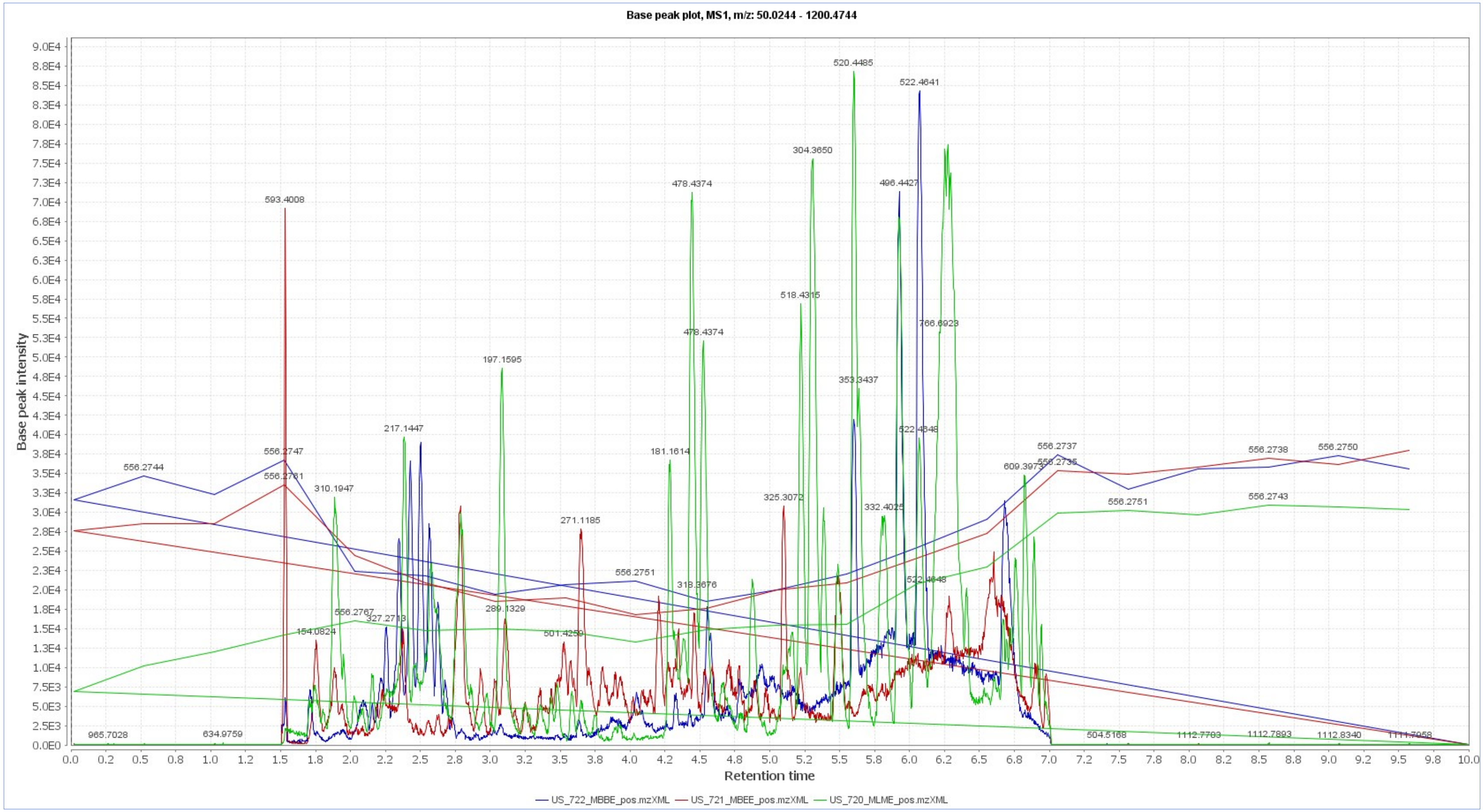

Figure S18: Total Ion chromatogram of different extracts of *Manilkara hexandra* (Roxb.) Dubard.

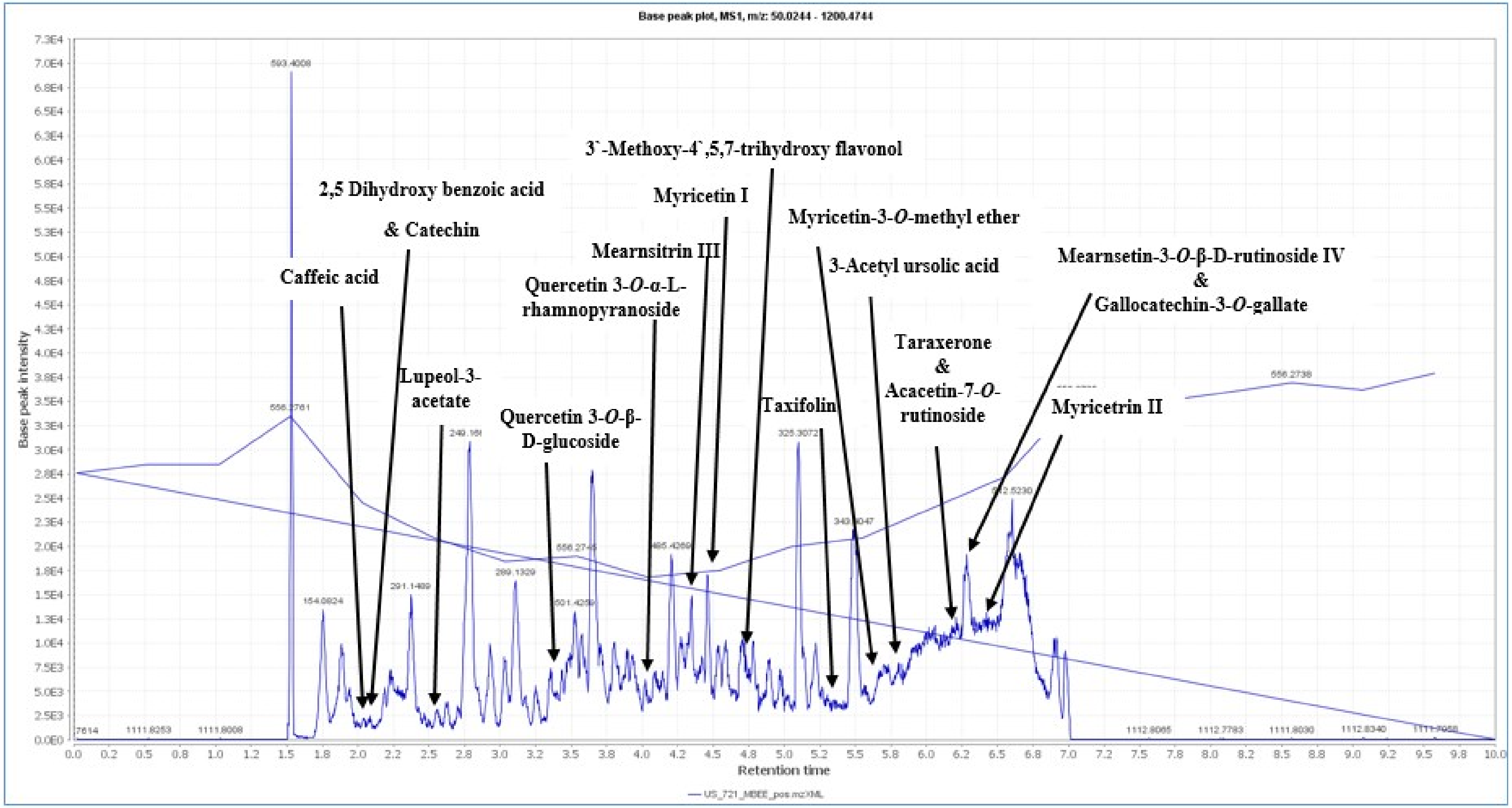

Figure S19: Total ion chromatogram of the ethyl acetate extract of *Manilkara hexandra* (Roxb.) Dubard bark

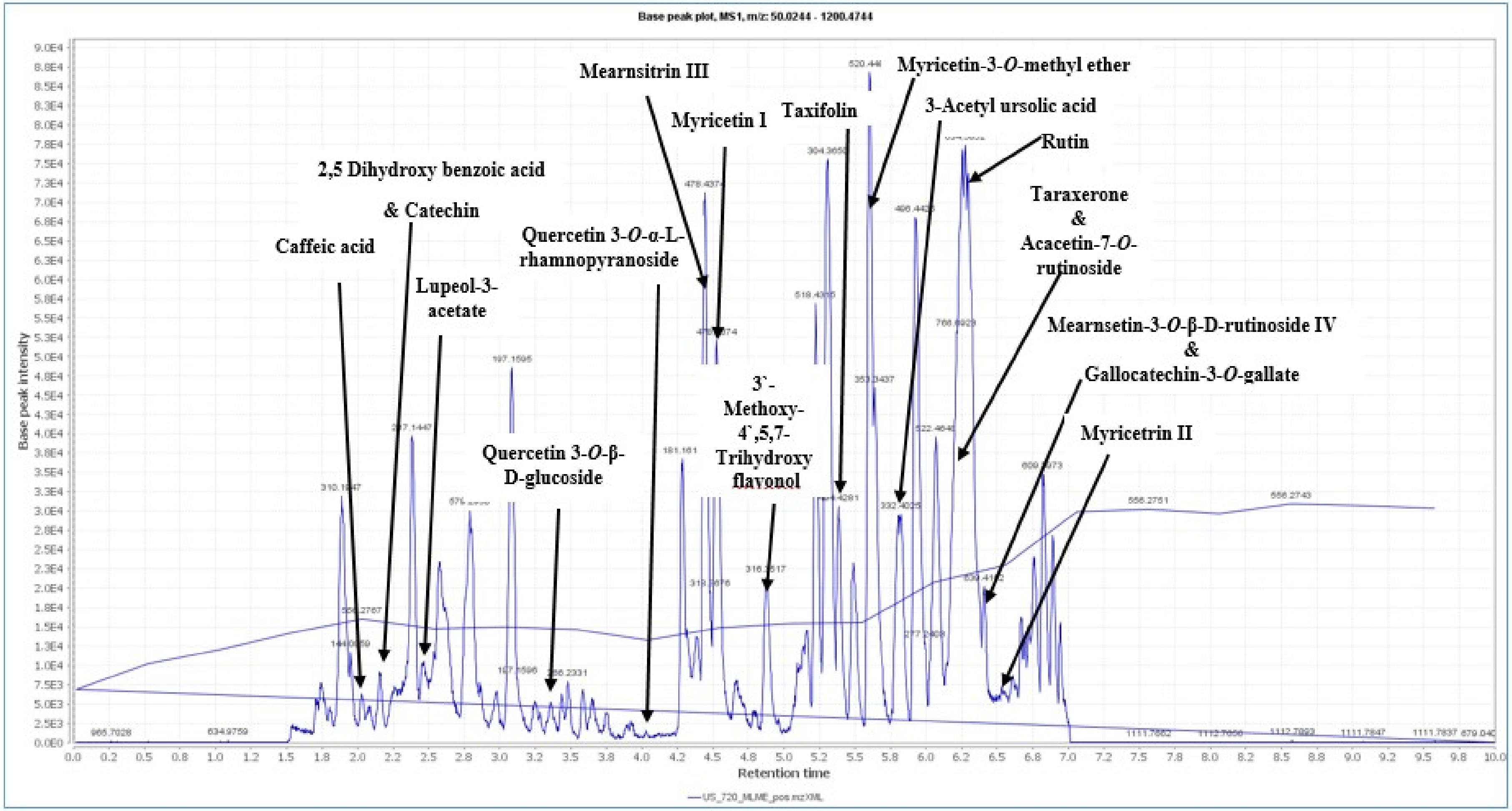

Figure S20: Total ion chromatogram of the methanol extract of *Manilkara hexandra* (Roxb.) Dubard leaves.
